# Supplementary material for: Rationale, development and feasibility of group antenatal care for immigrant women in Sweden: a study protocol for the Hooyo Project
Source: BMJ Open. 2019 Jul 31;9(7):e030314. doi: 10.1136/bmjopen-2019-030314 (PMC6677950; doi:10.1136/bmjopen-2019-030314)
Supplement: Author's manuscript [file bmjopen-2019-030314.draft_revisions.pdf]

BMJ Open is committed to open peer review. As part of this commitment we make the peer review history of every article we publish publicly available.

When an article is published we post the peer reviewers' comments and the authors' responses online. We also post the versions of the paper that were used during peer review. These are the versions that the peer review comments apply to.

The versions of the paper that follow are the versions that were submitted during the peer review process. They are not the versions of record or the final published versions. They should not be cited or distributed as the published version of this manuscript.

BMJ Open is an open access journal and the full, final, typeset and author-corrected version of record of the manuscript is available on our site with no access controls, subscription charges or pay-per-view fees (http://bmjopen.bmj.com).

If you have any questions on BMJ Open's open peer review process please email
info.bmjopen@bmj.com

BMJ Open

Rationale, development and feasibility of group antenatal care for immigrant women in Sweden: a study protocol for the Hooyo-project

Journal:	*BMJ Open*
Manuscript ID	bmjopen-2019-030314
Article Type:	Protocol
Date Submitted by the Author:	08-Mar-2019
Complete List of Authors:	Byrskog, Ulrika; Dalarna University, School of Education, Health and Social sciences<br>Ahrne, Malin ; Karolinska Institute, Department of Women's and Children's Health<br>Small, Rhonda; La Trobe University, Mother and Child Health Research<br>Andersson, Ewa; Karolinska Institute, Department of Women's and Children's Health<br>Essen, Birgitta; Uppsala University, Womens and Childrens Health<br>Adan, Aisha; Karolinska Institute, Department of Women's and Children's Health<br>Ahmed, Fardosa; Karolinska Institute, Department of Women's and Children's Health<br>Tesser, Karin; Antenatal Care Clinic, Domnarvet<br>Lidén, Yvonne; Antenatal Care Clinic<br>Israelsson, Monika; Antenatal Care Clinic<br>Åhman-Berndtsson, Anna; Antenatal Care Clinic, Domnarvet<br>Schytt, Erica; Centre for Clinical Research Dalarna-Uppsala University; Western Norway University of Applied Sciences, Faculty of Health and Social Sciences
Keywords:	Feasibility study, Immigrants, Somali-born women, Process evaluation, Midwives, Group antenatal Care

SCHOLARONE™
Manuscripts

Rationale, development and feasibility of group antenatal care for immigrant women in Sweden: a study protocol for the Hooyo-project

Ulrika Byrskog[a] uby@du.se

Malin Ahrne[b] malin.ahrne@ki.se

Rhonda Small[bc] R.Small@latrobe.edu.au

Ewa Andersson[b] Ewa.Andersson@ki.se

Birgitta Essén[d] birgitta.essen@kbh.uu.se

Aisha Adan[b] Shafeec08@hotmail.com

Fardosa Hassen Ahmed[b] fardosa.hassen.ahmed@ki.se

Karin Tesser[e] karin.tesser@Ltdalarna.se

Yvonne Lidén[f] yvonne.liden@sll.se

Monika Israelsson[f] monika.israelsson@sll.se

Anna Åhman Berndtsson[e] anna.ahman-berndtsson@Ltdalarna.se

Erica Schytt[bgh] Erica.Schytt@Ltdalarna.se

[a] School of Education, Health and Social Studies, Dalarna University, Falun, Sweden

[b] Department of Women's and Children's Health, Karolinska Institutet, Stockholm, Sweden

[c] Judith Lumley Centre, La Trobe University, Melbourne, Australia

[d] Women's and Children's Health, IMCH, Akademiska Hospital, Uppsala Sweden

[e] Antenatal Care Clinic, Domnarvet Borlänge, Sweden

[f] Antenatal Care Clinic, Spånga-Tensta, Sweden

[g] Centre for Clinical Research Dalarna-Uppsala University, Falun, Sweden

[h] Faculty of Health and Social Sciences, Western Norway University of Applied Sciences, Bergen, Norway

Corresponding author: Ulrika Byrskog uby@du.se
Dalarna University, School of Education, Health and Social Studies
791 88 Falun
Sweden

Word count excluding abstract, declarations, figure and references: 4924

For peer review only - http://bmjopen.bmj.com/site/about/guidelines.xhtml

ABSTRACT

Introduction Somali-born women comprise a large group of immigrant women of childbearing age in Sweden, with increased risks for perinatal morbidity and mortality and poor experiences of care, despite the goal of providing equitable health care for the entire population. Rethinking how care is provided to immigrant women may help to improve outcomes.

Overall aim To develop and test the acceptability, and immediate impacts of group antenatal care for Somali-born women residing in Sweden, in an effort to improve experiences of antenatal care, knowledge about childbearing and the Swedish health care system, emotional wellbeing and ultimately, pregnancy outcomes. This protocol describes the rationale, planning and development of the study.

Methods and analysis A feasibility study with focus group discussions, a historically controlled evaluation and process evaluation measures incorporated. Phase I includes needs assessment and development of contextual understanding. Phase II includes development of intervention and evaluation tools. Phase III is the implementation and evaluation of the intervention. In Phase IV, the process, feasibility and mechanisms of impact are investigated. Intervention development is based on theories of core values for quality of care, person-centred care, and group antenatal care. Study details have been considered in collaboration with a reference group, including relevant outcome measures: care satisfaction (Migrant Friendly Maternity Care Questionnaire), mental health (Edinburgh Postnatal Depression Scale), social support, childbirth fear, knowledge of Swedish maternity care, delivery outcomes.

Ethics and dissemination The study is approved by the Regional Ethical Review Board, Stockholm, Sweden. All participants receive information about the study and the voluntariness of participation and give written consent prior to enrolment. Participants will achieve a summary of the results in their language, and the findings will be disseminated at national and international conferences, through publications in peer-reviewed journals and seminars involving local stakeholders and practitioners, and via the project website.

Keywords: Feasibility study, immigrants, Somali-born women, midwives, group antenatal care, process evaluation

For peer review only - http://bmjopen.bmj.com/site/about/guidelines.xhtml

ARTICLE SUMMARY

strengths and limitations of this study

- The feasibility design of the study together with the nested process evaluation will contribute with valuable information for future controlled studies, and in the design of antenatal care interventions that target inequalities in health between immigrants and non-immigrants.
- Being a feasibility study, the study is neither designed nor powered to evaluate the effect of the new model of care.
- By emphasising dialogue, a person-centred approach and active participation of parents, midwives and bilingual research assistants throughout development and implementation, recruitment and retention are optimized and the study can hopefully contribute with a relevant, pragmatic and acceptable model of care that is replicable to other settings and groups with only minor adjustments.

INTRODUCTION

Sweden has a clearly stated goal of providing equitable health care for the whole population, which in recent decades has become increasingly diverse. Despite this, studies indicate that pregnancy outcomes among immigrant women are suboptimal compared with those of Swedish-born women(1). These health disparities point to the need for implementing and evaluating interventions to improve care for immigrant women and their families. This study protocol describes the development of an intervention to improve antenatal care (ANC) for Somali-born women and families giving birth in Sweden.

Somali-born women constitute one of the largest groups of immigrant women of childbearing age in Sweden(2), after more than two decades of political instability in Somalia. Of all immigrant women, they are known to be at highest risk of maternal- and perinatal morbidity and mortality both in Sweden(3-5) and internationally(6). In Sweden, later and lower attendance for antenatal care has been reported(7), and poorer experiences of care for labour and birth(8). Language difficulties and lack of familiarity with care systems, and discrimination and sub-optimal care are contributing factors(3, 9,10). Furthermore, higher rates of anaemia, insufficient weight gain and infants born

For peer review only - http://bmjopen.bmj.com/site/about/guidelines.xhtml

small for gestational age(7) indicate that Somali women are not reached by advice about prevention or treatment. Lower attendance at childbirth and parent education classes among immigrant women has also been demonstrated(11).

Few measures are taken in Sweden to reduce barriers to maternity care for immigrant women or to better meet their care needs. Furthermore, few studies have investigated how care might be designed in ways that are both attractive and acceptable to immigrant women and to midwives who are the main providers of care during pregnancy and childbirth. Respect, communication, community knowledge and care tailored to women's circumstances and needs have been proposed as central components in quality maternity care(12). In line with this, a systematic review in five countries highlighted that immigrant women want the same things from care as non-immigrant women; care should be of high quality and safe, with adequate information and support and attentive to individual needs, but immigrant women also consistently reported experiencing additional challenges, including communication difficulties, lack of familiarity with health care systems and discrimination(9).

Language barriers, marginalisation and prevailing cultural stereotypes pose challenges in designing, implementing and evaluating innovative care models including, rather than excluding, immigrants. These factors need to be carefully addressed before widespread change of new models of care are introduced. Participatory approaches have shown promising results(13-15) and reduces the risk of enhancing marginalization. Focus on communication and meaningful dialogue throughout intervention design, recruitment, data collection, implementation and evaluation is valuable (14).

In Sweden, the care of pregnant women during normal pregnancies is provided at community-based ANC clinics by registered midwives, based on national and regional guidelines(16). Included are 8-10 pregnancy check-ups free of charge, traditionally offered through individual appointments (30 minutes). Extra visits are booked and specialised medical referrals are made when needed. Parent education including preparation for childbirth and parenting is provided during the individual appointments or in groups/classes, mainly in Swedish and often focusing on first time parents. Partners are encouraged to participate.

One innovative approach is group antenatal care (gANC). This care model incorporates pregnancy assessments and parent education in small groups, with shorter individual appointments with the midwife at the beginning or end of the group session(17-19), while the rest of the group can

For peer review only - http://bmjopen.bmj.com/site/about/guidelines.xhtml

socialise. Results from studies in the US, Iran and Sweden report improved satisfaction with care(18, 20-23), improved iron taking, higher birthweight, lower caesarean section rates, more breastfeeding and earlier diagnosis of complications(22). Higher self-esteem and lower levels of stress, social conflicts and depression have been reported, as have more visits to ANC by fathers-to-be, increased support in social contacts with other parents, and reduced costs(17, 18, 22, 24-27), despite more time spent with the midwife throughout pregnancy. gANC thus appears to enhance women's agency, dialogue, time with the midwife and social interaction, and to improve women's views of their care and also some of their pregnancy outcomes. gANC may be especially valuable for specific groups of women, as seen for example in studies among young African-American women in the United States(25, 28) and among Karen refugee women in Australia(14).

The *Hooyo-project* ('mother' in Somali) is a gANC initiative developed in Sweden in response to the evidence about lack of equity in pregnancy care provision and poorer outcomes for Somali-born women. gANC has not been evaluated among immigrant women and families in Sweden, yet it may well be a model of care that can break down cultural and care barriers, enable better dialogue and understanding between caregivers and immigrant women and improve women's satisfaction with their care, especially if language support is provided(14).

The overall aim for this study is to develop and test the acceptability, and immediate impacts of gANC for Somali women residing in Sweden, in an effort to improve experiences of antenatal care, knowledge about childbearing and the Swedish health care system, emotional wellbeing and ultimately, pregnancy outcomes.

This includes

1) To establish with Somali-born women and antenatal caregivers the acceptability and feasibility of gANC and to develop an appropriate model in order to improve outcomes;

2) To establish appropriate recruitment and data collection procedures and outcome measures in consultation with the Somali community and with care providers in order to evaluate the new model; and

3) To implement and evaluate an agreed model of gANC in partnership with the Somali community and antenatal caregivers.

For peer review only - http://bmjopen.bmj.com/site/about/guidelines.xhtml

METHODS AND ANALYSIS

Design

A feasibility study including focus group discussions, a historically controlled evaluation and a process evaluation(29, 30). The intervention is implemented in two antenatal care clinics in mid-Sweden. The feasibility and piloting includes testing procedures for acceptability, estimating the likely rates of recruitment and retention of subjects, and the calculation of appropriate sample sizes for future controlled trials. Complex interventions that are adjusted to local, specific(31) or personal needs(32) is proposed to work better than completely standardised models(29). Key principles underpinning the study are therefore:

- Active involvement of Somali parents and midwives in needs assessment and care design
- Attention to language and contextual factors
- Flexibility in study methods to respond to issues as they arise
- A care model ready to continue or replicate after project ending with only minor adjustments.

Hooyo consists of four phases with process evaluation activities informing study progress. *Phase I* is the preparation phase, including needs assessment, the development of contextual understanding and building a logic model for the project. *Phase II* includes the development of the intervention and of the evaluation tool and *Phase III* is implementation and evaluation of the intervention using historical controls. In *Phase IV* the implementation process, the feasibility and the mechanisms of impact are evaluated, including contextual factors, with Moore's Process Evaluation of Complex Interventions as an overall framework(30).

Phase 1 – preparation phase

The purpose of Phase 1 was first to understand how current care was delivered and experienced and what changes women, partners and midwives thought might improve care, taking into account contextual factors that might impact on the development, structure and content of a new model. Second, to assess whether a language-supported group antenatal care model might be appropriate and acceptable to address the concerns raised.

For peer review only - http://bmjopen.bmj.com/site/about/guidelines.xhtml

Initial Dialogues and Choosing Study Settings

In the municipality for *site one,* the number of Somali immigrants had increased fivefold during the last decade and the ANC midwives had begun to re-think how they were providing care. Parallel, there was awareness of lower ANC attendance and adverse outcomes among child-bearing Somali-born women in Sweden(7), and a need for relevant explanations regarding ANC routines had been highlighted in interviews with Somali-born women(33). This prompted a dialogue between the principal investigator and the ANC midwives, outlining the possibility of evaluating an alternative model of care. The ANC clinic is located in a Primary Health Care Centre staffed by 10 midwives, within a public hospital located outside the city centre. It caters for approximately 75% of all pregnant women in the municipality and almost all of the pregnant immigrant women in the municipality, which in total has 50 000 residents. The majority of the Somali-born women visiting the clinic live in two areas situated approximately five kilometers away, necessitating use of a car or public transport.

In *site two*, the ANC clinic is located in a Family Health Centre in a suburb of the capital city. Close collaboration takes place between ANC midwives, social workers, child health nurses and the open play group located in the same building. Three ANC midwives are employed and the target area includes two residential areas with different socio-demographics; with primarily Swedish-born residents in one area and non-Swedish born residents or second generation immigrants in the other. Somali-born residents have lived in this area for more than 25 years and midwives reported that most Somali-born pregnant women and families were able to communicate in Swedish. Integration of families of different cultural backgrounds is actively encouraged at the clinic. Initial discussions revealed that the midwives believed group antenatal care for all women of mixed cultural background (with interpreters available if needed) would contribute positively to integration. As a result, it was agreed that integrated groups would be more appropriate and acceptable at this site.

Mechanisms for engagement: Reference Group and Bicultural Research Assistants

A project reference group was established comprising research team members, antenatal care midwives and representatives of the Somali community from both sites. Terms of reference were developed, including provision of advice to the research team on design of the intervention, appropriate recruitment processes and data collection methods; development of study questionnaires; networking with Somali associations and ANC clinics; as well as contributing to the interpretation and dissemination of results throughout the project period.

For peer review only - http://bmjopen.bmj.com/site/about/guidelines.xhtml

Bilingual Somali research assistants with health care backgrounds were employed full time in the project at site one. This enabled networking with Somali community members, bridging language gaps, input into questionnaire design, recruitment of participants, data collection and arranging and interpreting focus group discussions together with research team members. At site two, focus group discussions were facilitated by two Somali community workers, including recruitment and interpretation.

Participatory focus group discussions with Somali parents and antenatal care midwives

To develop understanding of current antenatal care and how it was experienced, Phase 1 included focus group discussions (FGDs) with Somali-born parents and with ANC midwives; presented in brief below and in Ahrne et al(34).

Parents were recruited using purposeful sampling. In total, 16 mothers and 13 fathers with varied length of stay in Sweden and recent experience (<2 years) of ANC were included. Seven ANC midwives were recruited purposefully from both sites and from one additional site where the midwives had previous experience of group antenatal care with Somali-born parents.

Three focus group discussions were held with Somali-born mothers, two with Somali-born fathers and three with ANC midwives. Emerging themes were highlighted by the researchers and crosschecked during the discussions with participants for accuracy(35, 36). Thematic analysis as described by Attride-Sterling(37) was used to categorize data from the FGDs.

Key findings informing intervention development

Challenges on system level and in the care encounter in striving for optimal ANC were identified; (34), alongside findings that could directly inform the intervention development. Instead of focusing on specific cultural or ethnic aspects of care the results indicated a need to maintain focus on diversity and individual needs. The need for forums for social interaction, and for dialogue which could bridge gaps between divergent health care systems, improve communication and reduce the risks for stereotyping was indicated. Responding to a wide range of health literacy needs, welcoming fathers and to safeguard an open atmosphere were other issues raised. Furthermore, practical issues such as optimal location and time points for ANC visits were discussed. The findings contributed with valuable input to the program theory and to the decisions regarding underpinning central principles for the coming intervention (Box 1), as well as for the development of structure and content in phase II.

For peer review only - http://bmjopen.bmj.com/site/about/guidelines.xhtml

- *Insert box 1 here* -

Box 1. Logic model of Hooyo including problem statements, conceptual framework and rationale, the Hooyo group antenatal care model, hypothesised mechanisms of effect and desired outcomes.

Phase II – Development of Intervention and Evaluation Tools

Developing group antenatal care

A review of literature and the experiences of team member EA had led us to investigate whether group antenatal care could be developed to improve outcomes for Somali-born women. The preliminary findings from the FGDs confirmed gANC as a possible alternative care model and was discussed in the reference group. Research team members participated in dialogues at each study site during these processes. The intervention development was characterized by constant modifications to fit local prerequisites at both sites, including local guidelines and routines at the ANC clinics, the needs in the areas and among the target groups; not all visible from the start.

Underpinning principles

Central findings in the FGDs were the desire for individualised care, need for forums for dialogue, to address variations of health literacy and at the same time answer up to the quest for moving beyond stereotypes based on ethnicity or "culture". This led us to focus on Person Centered Care (PCC)(38) as a base for the intervention, and on how this could be strengthened and encouraged through active dialogue in group based care. This was also in line with the ongoing implementation of PCC in the health system in general Sweden. In searching for a tool for this, Motivational Interviewing (MI) was proposed. The midwives had already received training in MI for use in individual appointments, and now MI *for groups* were added. Principles of person-centered care and MI encourage understanding the person as an individual, developing partnership and promoting self-efficacy, in which an active and open dialogue is central(38-40).

Addressing language and integration

To respond to the diverse needs identified at the two ANC clinics and to assess advantages and disadvantages with both homogeneous and heterogeneous groups regarding language and cultural background, it was decided that gANC would be offered to Somali-born women at site 1, and to *all* women attending the clinic, in integrated groups. At site 1, the need for a Somali interpreter at each session was anticipated. Since a finding in the FGD study was that continuity of Somali interpreters

For peer review only - http://bmjopen.bmj.com/site/about/guidelines.xhtml

known to the group had been a previous success factor, it was decided to engage two experienced interpreters who could alternate. At site two it was anticipated that the need for interpreters in different languages would vary and thus should be engaged when needed.

The intervention: Group Antenatal Care

The intervention consists of gANC which will be offered at both sites, modified to the needs in each site and group. Women will be allocated to a group with women at similar stages of pregnancy within a four-week gestational age span. From the second visit (gestational week 20-26) the women will receive group antenatal care. Each group will consist of 6-8 women and partners (or another support person). Frequency and number of sessions follow the national Swedish program for antenatal care, i.e. 8-9 appointments during a normal pregnancy. Each visit includes a group session for 1 hour, facilitated by one of two midwives assigned to the group, and with interpreter assistance. Although each group session has a focus on a theme related to pregnancy, birth or parenting in line with national recommendations(16), particular emphasis will be given to issues and questions raised by the participants. By applying a group design of care, we hypothesize that queries that for any reason are not verbalized in an individual encounter may be addressed in the dialogue between the midwife and other participants. Attached to the group discussion each woman has a 15-minute individual appointment with the responsible midwife for controls and private questions. In total the time spent with midwife at each visit will be approximately 75 minutes instead of 30 minutes as common during individual standard care. If medical or other reason, additional individual appointments can be booked.

Development of Evaluation Tools

Questionnaires

Questionnaires for data collection from participating women were developed and modified from the Migrant Friendly Maternity Care Questionnaire (MFMCQ)(41) to fit the purpose of the study. It also included modified questions from the Cambridge Worry Scale which is available in English and Swedish(42), and the Edinburgh Postnatal Depression Scale (EPDS) which is available in English, Swedish and Somali(43). The questionnaires were developed in English by the research team and translated to Swedish by one of the bilingual team members and thereafter cross-checked by another bilingual researcher in the team. Thereafter the questionnaire was translated into Somali by a professional Somali translator, fluent in English, Swedish and Somali, and finally crosschecked by a Somali-speaking research assistant. In each step of the process the questionnaire

For peer review only - http://bmjopen.bmj.com/site/about/guidelines.xhtml

was pilot-tested with Somali-born women for relevance and understanding with adjustments made accordingly. During piloting it was clear that some of the questions and response alternatives in the EPDS-scale were complicated in their phrasing. Although the scale is translated into Somali, no validation study has to date been published. A decision was made to include EPDS in its present form with the research assistant being ready to explain in plain language when needed. Parallel a validation process for the Somali version including think-aloud interviews(44) with Somali-born women has been commenced, in order to contribute to evidence about use of the EPDS in Somali.

Preparing for implementation

Awareness raising

To create awareness and interest in the intervention, Somali and other community networks are engaged in different ways. Information is spread by participants in the reference group, by researchers and research assistants one-to-one and in small groups gathering Somali-born women. A brief pamphlet in Swedish and Somali is produced and distributed among women, in Somali community associations, open play-schools and activity centers and as a poster in the waiting rooms at participating ANC clinics.

Training of midwives and interpreters

Areas to be covered in a preparatory training workshop for midwives before intervention start were identified through findings in the FGDs, in dialogue with the midwives at both sites and in reference group meetings. The research team then tailored a 1 ½ day workshop for all midwives at both sites, held prior to intervention commencement. In response to the needs expressed, the main focus was group processes including person-centering(38) and motivational interviewing (MI) in groups(40). A two-day follow-up session is planned during the intervention period to enable feedback on implementation and address any issues arising.

The interpreters' preparations included two hours of information regarding the structure of the ANC care system, introduction to the Hooyo-project emphasising dialogue and MI-principles, participation in meetings with involved midwives and taking part of a written manual.

Development of material for midwives leading group sessions

A manual comprising information and suggestions for content and structure of group sessions is developed, which the midwives are free to use and adjust according to the needs in the groups. An overview of available evidence based material related to pregnancy and birth led us to recommend

For peer review only - http://bmjopen.bmj.com/site/about/guidelines.xhtml

the use of existing material, as it is available free of charge and often in different languages. This added to the sustainability and cost-effectiveness of the intervention, and makes it easier to duplicate. A study-specific homepage was created, comprising the same information and support as in the written manual.

Phase 3 – Controlled evaluation of the intervention

Participants and recruitment

In both sites, primiparous and multiparous Somali-born women are recruited to the study. Recruitment and retention of participants to clinical studies is a well-documented challenge in research across languages and cultures(45). We therefore thoroughly planned how to optimize the recruitment and information processes in relation to eligible women and to adjust this process to local prerequisites in both sites. The Somali-speaking research assistant is based in site 1 where it is considered most needed, and where the major part of the recruitment is performed. In site 2, team-member MA work with recruitment, information and data collection, supported by the Somali-speaking research assistant in site 1. All pregnant Somali-born women attending the two ANC clinics receive brief information about the study from the midwives during their first visit and if they agree to receive further information, they are then contacted by the research assistant or MA after the first ANC visit, either by telephone or face to face for further oral and written information in Somali, English or Swedish, as needed. *Inclusion criteria* are being born in Somalia and <25 weeks of gestation. *Exclusion criterion* is a health condition preventing participation in group antenatal care, i.e. severe mental health condition or if the pregnancy care were to be transferred to obstetric specialist clinic. During the first 18 months of the study period, recruited women were included in the control group receiving standard individual care and in the following 18 months women are included in the intervention group.

Outcome measures

Primary outcomes are women's overall ratings of antenatal care and emotional wellbeing, assessed by the core question "When thinking about your overall experience of antenatal care - in general, have you been happy with the care that you have received?" with response alternatives *always, mostly, sometimes, rarely and never*. The question is modified from the Migrant Friendly Maternity Care Questionnaire (MFMCQ)(41). Emotional wellbeing is assessed by the Edinburgh Postnatal Depression Scale (EPDS), available in English, Swedish and Somali(43).

For peer review only - http://bmjopen.bmj.com/site/about/guidelines.xhtml

Secondary outcomes are adequate number of visits measured by the Adequacy of Prenatal Care Utilization Index (APCUI), i.e. expected number of visits in relation to actual number adjusted for gestational week at booking visit and delivery(46), social support during pregnancy by slightly modified questions from the Pregnancy Risk Assessment Monitoring System (PRAMS)(47) and worries about the upcoming birth retrieved from the Cambridge Worry Scale(42). Knowledge about danger signs and where to seek health care if certain symptoms arise are assessed with multiple choice questions, such as "Now at the end of your pregnancy, where would you turn if a bleeding from vagina would occur? When would you seek care?". From the patient record, the following are retrieved: estimated time spent with the antenatal midwife, gestational age at the first ANC visit, lowest measurements of Haemoglobin and S-Ferritin, weight gain, attendance at parent education, mode of birth, interventions (induction of labour, pain relief including epidural, oxytocin), blood loss, diagnoses (mother) and gestational age, birth weight, Apgar score, umbilical cord pH, and the need for neonatal intensive care (infant).

Data collection for the intervention

Questionnaires

Questionnaire data are collected at three time points (Q1, Q2, Q3). The baseline measurement (Q1) is conducted after recruitment, i.e. before gestational week 25. Women are contacted for a face-to-face or telephone structured interview in Somali, Swedish or English at a place of their choice. Q1 includes questions on background (education, occupation), migration (time of residence, reason for migration, migration status, language) and social support, psychological and physical wellbeing, knowledge of danger signs and the health system, and expectations about the up-coming birth. Data for Q2 are collected in late pregnancy, after gestational week 35, in the same manner. Q2 includes questions on experience of care and information during the ongoing pregnancy, knowledge of the health system, pregnancy and birth, social support, and psychological and physical wellbeing and expectations for the upcoming birth. Q3 is administered two months postpartum and includes questions regarding the overall experience of pregnancy, ANC, birth and psychological and physical wellbeing. After each interview a small gift is offered as a token of appreciation for women's participation.

Medical records

From patient medical records, data for secondary outcome measurement will be collected. This includes data related pregnancy, labour and birth and maternal and infant outcomes.

For peer review only - http://bmjopen.bmj.com/site/about/guidelines.xhtml

For peer review only

Sample size issues

The study is a feasibility study(29, 48), not designed to have the power to detect differences on the primary outcomes. Our main purpose is rather to develop and test the feasibility of the intervention and study details and in so doing also provide information to inform sample size calculations for possible future controlled trials. Based on available birth rate data we estimate being able to include 100 women in the control group and 100 women in the intervention group during the study period. Differences between groups will be described as odds ratios and 95% confidence intervals after estimation by means of logistic regression analyses. Comparison of means will be undertaken using t-tests where data are normally distributed, or medians compared using Mann-Whitney U tests if not.

Phase 4 – Process evaluation

A process evaluation using mixed methods approach is conducted throughout the study (30). The process evaluation will address whether the intervention was carried out according to plan, what was achieved, if and what adaptations were needed, who was reached by the intervention, any unexpected events and to understand the mechanisms of impact and what mediated these mechanisms. The process evaluation will also address contextual factors(30).

Process evaluation data collection and measures

Data collection for the process evaluation(30) is nested in the different phases, supported by different measurement tools.

A session Checklist for completion by the midwife after each group session covers topics, material used, number of participating women and support people, group dialogue and the midwife's brief view of each session. The checklist was developed by the research team, discussed with the reference group and, after adjustments, presented to all included midwives for further modifications, which were addressed by the researchers before intervention start.

Participant observations during the group sessions will contribute with data regarding how delivery was achieved and the mechanisms of impact by describing dynamics, dialogue and active participation during the intervention. A protocol for observations during group sessions is being developed by the research team, focusing on group mechanisms, dialogue and interaction, based on PPC and MI principles. This will provide the base for participant observations by the principal investigator during a number of randomly selected group sessions.

For peer review only - http://bmjopen.bmj.com/site/about/guidelines.xhtml

Participant experiences of group antenatal care. Questionnaire data for participating women and qualitative data collected from midwives, partners, interpreters and head of departments will describe responses to and interactions with the intervention and with their views regarding the feasibility of the intervention and mechanisms of impact. Women's experiences of content, structure and feasibility of gANC will be assessed by means of the *follow-up questionnaire* (Q3) at 2 months postpartum. Midwives' experiences of the same, and their perceptions regarding mechanisms of impact will be collected through *qualitative FGDs or interviews*. Partners' and interpreters' views will be collected through *qualitative individual interviews* upon completion of participation. FGD and interview data will be collected, through a topic guide with open-ended questions developed by the research team based on Moore's process evaluation framework (30).

Field notes taken by the researchers in dialogue with research assistants, involved midwives and heads of departments will support in describing the choices and decisions made during the implementation process.

DISCUSSION

The study is an attempt to address some of the health and care disparities in the Swedish health care system. The feasibility design of the study(29) together with the nested process evaluation(30) will contribute with valuable information for future controlled studies, as well as in the design of antenatal care interventions that target inequalities in health between immigrants and non-immigrants. The study will provide guidance on acceptability of this model of care among Somali-born women, their partners and midwives in Sweden, and on the recruitment and data collection from responders who often are excluded in research due to communication barriers or marginalization. The study will also indicate whether gANC may improve the experiences of antenatal care and therefore would be appropriate for trials with other migrant groups or women with other joint health or life style factors.

For the midwives, the intervention provides a platform for internal reflections and dialogue about making the care accessible and improved. This strengthens the justification, since care provision is always an interplay between care-givers and care-receivers. The early inclusion of midwives' and participants' views provided space to shape the planned intervention in relevant ways in terms of content, structure and underlying core principles(32). A challenge is to develop an acceptable model

For peer review only - http://bmjopen.bmj.com/site/about/guidelines.xhtml

of care addressing diverse needs of the care-receiving group and local conditions while ensuring that medical and public health guidelines are fulfilled. By emphasising dialogue, a person-centred approach and the active participation of women, partners and midwives throughout development and implementation of the intervention, we aim to develop a relevant, pragmatic and acceptable model of care, and one that is replicable to other settings and groups with only minor adjustments.

ETHICS AND DISSEMINATION

The study is approved by the Regional Ethical Review Board, Stockholm, Sweden, 2015-12-04, Dnr 2015/1703-31/1. All participants are offered information about the study, informed about the voluntary nature of participation in detail and give their written consent prior to enrolment.

The findings of the study will be disseminated at relevant national and international conferences and through publications in peer-reviewed journals. Seminars involving local stakeholders in the communities, county council representatives and practitioners will further provide a platform for dissemination and reflections about lessons learned at the end of the project. All participants in the study will achieve a summary of the results, and the findings of the study will be presented via the Hooyo-project website https://ki.se/kbh/modrahalsovard-for-utlandsfodda-kvinnor-hooyo-projektet.

DECLARATIONS

Acknowledgements
The authors would like to thank the midwives, members of the Hooyo reference group and all Somali-born women and men contributing in the preparation phase of the study.

Consent for publication

Not applicable.

Availability of data and material
The dataset generated and analysed during the preparation phase of the current study is not publicly available due to restrictions based on Swedish law safeguarding individual privacy. It may be available upon reasonable request.

Competing interests

The authors declare that they have no competing interests.

For peer review only - http://bmjopen.bmj.com/site/about/guidelines.xhtml

Funding

This study has received funding from the Swedish Research Council [grant number 2015-02470], Forte [grant number 2016-00957] and the Doctoral School in Health Care Sciences, Karolinska Institutet [grant number 2-144/2016]. The funding bodies have played no role in the design of the study, nor in data collection, analysis and interpretation of data or in writing the manuscript.

Author statement

ES, RS, and EA initiated the study. UB, ES, RS, EA MA, BE, AA, FH, KT, YL, MI and AB contributed to planning and design. UB, MA, ES, RS, EA AA and FH developed questionnaires and topic guides. UB drafted the manuscript, MA, ES, RS, UB BE, AA, FH, KT, YL, MI and AB revised it. All authors read and approved the final manuscript.

REFERENCES

1 National Board of Health and Welfare. Socioekonomiska faktorers påverkan på kvinnors och barns hälsa efter förlossning. Stockholm: National Board of Health and Welfare; 2016.

2 Statistics Sweden [Internet]. [Accessed 2018 Aug 20]. Available from: http://www.statistikdatabasen.scb.se/.

3 Esscher A, Binder-Finnema P, Bodker B, et al. Suboptimal care and maternal mortality among foreign-born women in Sweden: maternal death audit with application of the 'migration three delays' model. *BMC Pregnancy Childbirth* 2014;14:141.

4 Essen B, Bodker B, Sjoberg NO, et al. Are some perinatal deaths in immigrant groups linked to suboptimal perinatal care services? *BJOG* 2002;109(6):677-82.

5 Essen B, Hanson BS, Ostergren PO, et al. Increased perinatal mortality among sub-Saharan immigrants in a city-population in Sweden. *Acta Obstet Gynecol Scand* 2000;79(9):737-43.

6 Small R, Gagnon A, Gissler M, et al. Somali women and their pregnancy outcomes postmigration: data from six receiving countries. *BJOG* 2008;115(13):1630-40.

7 Rassjo EB, Byrskog U, Samir R, et al. Somali women's use of maternity health services and the outcome of their pregnancies: a descriptive study comparing Somali immigrants with native-born Swedish women. *Sex Reprod Healthc* 2013;4(3):99-106.

8 Berggren V, Bergstrom S, Edberg AK. Being different and vulnerable: experiences of immigrant African women who have been circumcised and sought maternity care in Sweden. *J Transcult Nurs* 2006;17(1):50-7.

For peer review only - http://bmjopen.bmj.com/site/about/guidelines.xhtml

9 Small R, Roth C, Raval M, et al. Immigrant and non-immigrant women's experiences of maternity care: a systematic and comparative review of studies in five countries. *BMC Pregnancy Childbirth* 2014;14:152.

10 Binder P, Borne Y, Johnsdotter S, et al. Shared Language Is Essential: Communication in a Multiethnic Obstetric Care Setting. *J Healthc Commun* 2012;17(10):1171-86.

11 Fabian H, Radestad I, Rodriguez A, et al. Women with non-Swedish speaking background and their children: a longitudinal study of uptake of care and maternal and child health. *Acta paediatrica* 2008;97(12):1721-8.

12 Renfrew MJ, McFadden A, Bastos MH, et al. Midwifery and quality care: findings from a new evidence-informed framework for maternal and newborn care. *Lancet* 2014;384(9948):1129-45.

13 Osman F, Salari R, Klingberg-Allvin M, Schon UK, et al. Effects of a culturally tailored parenting support programme in Somali-born parents' mental health and sense of competence in parenting: a randomised controlled trial. *BMJ open* 2017;7(12):e017600.

14 Riggs E, Muyeen S, Brown S, , et al. Cultural safety and belonging for refugee background women attending group pregnancy care: An Australian qualitative study. *Birth* 2017;44(2):145-52.

15 Riggs E, Yelland J, Szwarc J, et al. Promoting the inclusion of Afghan women and men in research: reflections from research and community partners involved in implementing a 'proof of concept' project. *Int J Equity Health* 2015;14:13.

16 The Swedish Society of Obstetrics and Gynecology (SFOG) and The Swedish Association of Midwives. Mödrahälsovård, Sexuell och Reproduktiv Hälsa. 2016; ARG report 76.

17 Lathrop B. A systematic review comparing group prenatal care to traditional prenatal care. *Nurs Womens Health* 2013;17(2):118-30.

18 Andersson E, Christensson K, Hildingsson I. Mothers' satisfaction with group antenatal care versus individual antenatal care - a clinical trial. *Sex Reprod Healthc* 2013;4(3):113-20.

19 Hunter L, Da Motta G, McCourt C, et al. `It makes sense and it works': Maternity care providers' perspectives on the feasibility of a group antenatal care model (Pregnancy Circles). *Midwifery* 2018;66:56-63.

20 Ickovics JR, Kershaw TS, Westdahl C, et al. Group prenatal care and perinatal outcomes: a randomized controlled trial. *Obstet Gynecol* 2007;110(2 Pt 1):330-9.

21 Jafari F, Eftekhar H, Mohammad K, et al. Does Group Prenatal Care Affect Satisfaction And Prenatal Care Utilization in Iranian Pregnant Women? *Iran J Public Health* 2010;39(2):52-62.

For peer review only - http://bmjopen.bmj.com/site/about/guidelines.xhtml

22 Jafari F, Eftekhar H, Fotouhi A, Mohammad K, Hantoushzadeh S. Comparison of maternal and neonatal outcomes of group versus individual prenatal care: a new experience in Iran. *Health Care Women Int* 2010;31(7):571-84.

23 Andersson E, Christensson K, Hildingsson I. Parents' experiences and perceptions of group-based antenatal care in four clinics in Sweden. *Midwifery* 2012;28(4):502-8.

24 Wedin K, Molin J, Crang Svalenius EL. Group antenatal care: new pedagogic method for antenatal care--a pilot study. *Midwifery* 2010;26(4):389-93.

25 Ickovics JR, Kershaw TS, Westdahl C, et al. Group prenatal care and perinatal outcomes - A randomized controlled trial. *Obstetrics and Gynecology* 2007;110(2):330-9.

26 Ickovics JR, Reed E, Magriples U, et al. Effects of group prenatal care on psychosocial risk in pregnancy: Results from a randomised controlled trial. *Psychology & health* 2011;26(2):235-50.

27 Andersson E, Small R. Fathers' satisfaction with two different models of antenatal care in Sweden - Findings from a quasi-experimental study. *Midwifery* 2017;50:201-7.

28 Ickovics JR, Earnshaw V, Lewis JB, et al. Cluster Randomized Controlled Trial of Group Prenatal Care: Perinatal Outcomes Among Adolescents in New York City Health Centers. *Am J Public Health* 2016;106(2):359-65.

29 Craig P, Dieppe P, Macintyre S, et al. Developing and evaluating complex interventions: the new Medical Research Council guidance. *Int J Nurs Stud* 2013;50(5):587-92.

30 Moore GF, Audrey S, Barker M, et al. Process evaluation of complex interventions: Medical Research Council guidance. *BMJ* 2015;350:h1258.

31 Campbell NC, Murray E, Darbyshire J, et al. Designing and evaluating complex interventions to improve health care. *BMJ* 2007;334(7591):455-9.

32 Yardley L, Morrison L, Bradbury K, et al. The person-based approach to intervention development: application to digital health-related behavior change interventions. *J Med Internet Res* 2015;17(1):e30.

33 Byrskog U, Essen B, Olsson P, et al. 'Moving on' Violence, wellbeing and questions about violence in antenatal care encounters. A qualitative study with Somali-born refugees in Sweden. *Midwifery* 2016;40:10-7.

34 Ahrne M, Adan A, Schytt E et al. Antenatal care for Somali born women in Sweden – perspectives from mothers, fathers and midwives. 1st World Congress on Migration, Etnicity, Race and Health: Diversity and Health. 2018 May 17-19; Edinburgh [Oral presentation, abstract published]. *Eur J Public Health* 2018:28(Suppl 1):cky047.252.

For peer review only - http://bmjopen.bmj.com/site/about/guidelines.xhtml

35 Lionis C, Papadakaki M, Saridaki A, et al. Engaging migrants and other stakeholders to improve communication in cross-cultural consultation in primary care: a theoretically informed participatory study. *BMJ open* 2016;6(7):e010822.

36 Johnson CE, Ali SA, Shipp MP. Building community-based participatory research partnerships with a Somali refugee community. *Am J Prev Med* 2009;37(6 Suppl 1):S230-6.

37 Attride-Stirling J. Thematic networks: an analytic tool for qualitative research. *Qualitative Research* 2001;1(3):385-405.

38 Ekman I, Swedberg K, Taft C, et al. Person-centered care - ready for prime time. *Eur J Cardiovasc Nurs* 2011;10(4):248-51.

39 Lundahl B, Moleni T, Burke BL, et al. Motivational interviewing in medical care settings: a systematic review and meta-analysis of randomized controlled trials. *Patient Educ Couns* 2013;93(2):157-68.

40 Wagner CC. Motivational Interviewing in Groups. New York: Guilford Publications; 2013.

41 Gagnon AJ, DeBruyn R, Essen B, et al. Development of the Migrant Friendly Maternity Care Questionnaire (MFMCQ) for migrants to Western societies: an international Delphi consensus process. *BMC Pregnancy Childbirth* 2014;14:200.

42 Green JM, Kafetsios K, Statham HE, et al. Factor structure, validity and reliability of the Cambridge Worry Scale in a pregnant population. *J Health Psychol* 2003;8(6):753-64.

43 Cox J, Holden J, Henshaw C. Perinatal Mental Health. The Edinburgh Postnatal Depression Scale (EPDS) Manual. 2nd ed: The Royal College of Psychiatrists; 2012.

44 Sudman S, Bradburn NM, Schwarz N. Thinking about answers: the application of cognitive processes to survey methodology. San Francisco: Jossey-Bass Publishers; 1996.

45 Liamputtong P. Performing Qualtiative Cross-cultural Research. Cambridge: Cambridge University Press; 2010.

46 Colon-Burgos JF, Colon-Jordan HM, Reyes-Ortiz VE, et al. Disparities and barriers encountered by immigrant Dominican mothers accessing prenatal care services in Puerto Rico. *J Immigr Minor Health* 2014;16(4):646-51.

47 Center for Disease Control and Prevention. Pregnancy Risk Assessment Monitoring System (PRAMS) [Internet]. [Accessed 2016 April 2]. Available from: http://www.cdc.gov/prams/.

48 National Institute for Health Research. Feasability and Pilot Studies: a guide for NIHR Research Design Service advisors. United Kingdom: National Institute for Health Research; 2016.

For peer review only - http://bmjopen.bmj.com/site/about/guidelines.xhtml

For peer review only

For peer review only - http://bmjopen.bmj.com/site/about/guidelines.xhtml

Problem statement	Conceptual framework and rationale	Hooyo: A group approach to improving ANC	Hypothesised mechanisms of effect	Desired outcomes
**Current ANC in Sweden may not provide equitable care for Somali-born women:**<br>• Lower participation in antenatal care<br>• Poorer birth outcomes<br>• Communication difficulties<br>• Lack of familiarity with Swedish antenatal care structures<br>• Lower attendance in parental education<br>• Negative attitudes and suboptimal care<br>**Initial FGDs with Somali-born parents/ANC midwives highlight need for:**<br>• Improved communication and dialogue<br>• Bridging gaps between divergent health literacy knowledge<br>• Care free from generalisa-tions, tailored to individual needs<br>• Clearly described expecta-tions regarding partner's role	**Core values for quality care:** respect, communication, community knowledge and understanding<br><br>**Person-centred care** to identify and address women's individual needs<br>**Continuity of care** for positive care experiences and health outcomes<br><br>**Group antenatal care** a promising alternative to individual visits:<br>• More positive views of care<br>• Some positive impacts on birth outcomes<br>• More time with midwives and more comprehensive parental education<br>• In Sweden studied with Swedish-speaking groups only<br><br>**Key underpinning principles**:<br>• Active involvement of Somali parents/ midwives in needs assessment and care design<br>• Attention to language and contextual factors<br>• Flexibility in study methods to respond to issues as they arise<br>• A care model ready to continue or replicate after project ending with minor adjustments	**Language supported group antenatal care,** involving<br>• 8-9 group sessions 1 1/2 hours with 6-8 women (partners welcome) from gest. week 24<br>• Facilitated by two midwives assisted by interpreter<br>• Brief individual midwife check-ups incorporated<br>• Childbirth/parenting themes with focus on dialogue and discussion<br>• Motivational interviewing for groups as a vehicle for focusing care on women's needs<br>• Adjustments based on local needs at each site:<br>*Site 1:* Groups specifically for Somali-born<br>*Site 2*: Groups with diverse backgrounds and languages	**Interpreter-supported group dialogue facilitated by midwives will result in**<br>• Improved communication →better suited care<br>• More time for discussions →mutual understandings in views around childbirth and health promotion → strategies for improving outcomes<br>• An additional arena for social contact and support →increased well-being<br>• Combining pregnancy check-ups with groups →motivation for attending ANC, and parental education<br>• Common language/ background → understanding and empower women to raise voices in having needs addressed<br>• Mixed groups →integration and understanding through cross-language/culture interactions	**Women:**<br>• Happier with the ANC<br>• More confident in and knowledgeable about the pregnancies<br>• Improved wellbeing<br>• Improved attendance at antenatal care visits<br>• Improved uptake of health advice<br>• Ultimately; improved pregnancy outcomes<br><br>**Partners:**<br>• Feeling welcomed and included<br>• Increased understanding of expectations<br><br>**Midwives**:<br>• Improved understanding of womens needs<br>• Feel better able to share health knowledge in meaningful ways<br>• Provide more supportive, non-judgemental care<br>• Positive about benefits of group care

Box 1. Logic model of Hooyo including problem statements, conceptual framework and rationale, the Hooyo group antenatal care model, hypothesised mechanisms of effect and desired outcomes.

For peer review only

For peer review only - http://bmjopen.bmj.com/site/about/guidelines.xhtml

BMJ Open

Rationale, development and feasibility of group antenatal care for immigrant women in Sweden: a study protocol for the Hooyo-project

Journal:	*BMJ Open*
Manuscript ID	bmjopen-2019-030314.R1
Article Type:	Protocol
Date Submitted by the Author:	15-May-2019
Complete List of Authors:	Byrskog, Ulrika; Dalarna University, School of Education, Health and Social sciences<br>Ahrne, Malin ; Karolinska Institute, Department of Women's and Children's Health<br>Small, Rhonda; La Trobe University, Mother and Child Health Research<br>Andersson, Ewa; Karolinska Institute, Department of Women's and Children's Health<br>Essen, Birgitta; Uppsala University, Womens and Childrens Health<br>Adan, Aisha; Karolinska Institute, Department of Women's and Children's Health<br>Ahmed, Fardosa; Karolinska Institute, Department of Women's and Children's Health<br>Tesser, Karin; Antenatal Care Clinic, Domnarvet<br>Lidén, Yvonne; Antenatal Care Clinic<br>Israelsson, Monika; Antenatal Care Clinic<br>Åhman-Berndtsson, Anna; Antenatal Care Clinic, Domnarvet<br>Schytt, Erica; Centre for Clinical Research Dalarna-Uppsala University; Western Norway University of Applied Sciences, Faculty of Health and Social Sciences
**Primary Subject Heading**:	Public health
Secondary Subject Heading:	Public health, Health services research, Obstetrics and gynaecology, Research methods, Patient-centred medicine
Keywords:	Feasibility study, Immigrants, Somali-born women, Process evaluation, Midwives, Group antenatal Care

SCHOLARONE™
Manuscripts

Rationale, development and feasibility of group antenatal care for immigrant women in Sweden: a study protocol for the Hooyo-project

Ulrika Byrskog[a] uby@du.se

Malin Ahrne[b] malin.ahrne@ki.se

Rhonda Small[bc] R.Small@latrobe.edu.au

Ewa Andersson[b] Ewa.Andersson@ki.se

Birgitta Essén[d] birgitta.essen@kbh.uu.se

Aisha Adan[b] Shafeec08@hotmail.com

Fardosa Hassen Ahmed[b] fardosa.hassen.ahmed@ki.se

Karin Tesser[e] karin.tesser@Ltdalarna.se

Yvonne Lidén[f] yvonne.liden@sll.se

Monika Israelsson[f] monika.israelsson@sll.se

Anna Åhman Berndtsson[e] anna.ahman-berndtsson@Ltdalarna.se

Erica Schytt[bgh] Erica.Schytt@Ltdalarna.se

[a] School of Education, Health and Social Studies, Dalarna University, Falun, Sweden

[b] Department of Women's and Children's Health, Karolinska Institutet, Stockholm, Sweden

[c] Judith Lumley Centre, La Trobe University, Melbourne, Australia

[d] Women's and Children's Health, IMCH, Akademiska Hospital, Uppsala Sweden

[e] Antenatal Care Clinic, Domnarvet Borlänge, Sweden

[f] Antenatal Care Clinic, Spånga-Tensta, Sweden

[g] Centre for Clinical Research Dalarna-Uppsala University, Falun, Sweden

[h] Faculty of Health and Social Sciences, Western Norway University of Applied Sciences, Bergen, Norway

Corresponding author: Ulrika Byrskog uby@du.se
Dalarna University, School of Education, Health and Social Studies
791 88 Falun
Sweden

For peer review only

For peer review only - http://bmjopen.bmj.com/site/about/guidelines.xhtml

ABSTRACT

Introduction Somali-born women comprise a large group of immigrant women of childbearing age in Sweden, with increased risks for perinatal morbidity and mortality and poor experiences of care, despite the goal of providing equitable health care for the entire population. Rethinking how care is provided may help to improve outcomes.

Overall aim To develop and test the acceptability, feasibility and immediate impacts of group antenatal care for Somali-born immigrant women, in an effort to improve experiences of antenatal care, knowledge about childbearing and the Swedish health care system, emotional wellbeing and ultimately, pregnancy outcomes. This protocol describes the rationale, planning and development of the study.

Methods and analysis An intervention development and feasibility study. Phase 1 includes needs assessment and development of contextual understanding using focus group discussions. In Phase 2 the intervention and evaluation tools, based on core values for quality care and person-centred care, are developed. Phase 3 includes the historically controlled evaluation in which relevant outcome measures are compared for women receiving individual care (2016-2018) and women receiving group antenatal care (2018-2019): care satisfaction (Migrant Friendly Maternity Care Questionnaire), emotional wellbeing (Edinburgh Postnatal Depression Scale), social support, childbirth fear, knowledge of Swedish maternity care, delivery outcomes. Phase 4, the process evaluation, investigate process, feasibility and mechanisms of impact using field notes, observations, interviews and questionnaires. All phases are conducted in collaboration with a stakeholder reference group.

Ethics and dissemination The study is approved by the Regional Ethical Review Board, Stockholm, Sweden. Participants receive information about the study and their right to decline/ withdraw without consequences. Consent is given prior to enrolment. Findings will be disseminated at antenatal care units, national/international conferences, through publications in peer-reviewed journals, seminars involving stakeholders, practitioners, community and via the project website. Participating women will receive a summary of results in their language.

Keywords: Feasibility study, immigrants, Somali-born women, midwives, group antenatal care, process evaluation

For peer review only

ARTICLE SUMMARY

Strengths and limitations of this study

- The feasibility design of the study together with the nested process evaluation will contribute valuable information for future controlled studies, and in the design of antenatal care interventions that target inequalities in health between immigrants and non-immigrants.
- By emphasising dialogue, a person-centred approach and active participation of parents, midwives and bilingual research assistants throughout development and implementation, recruitment and retention are optimised and a relevant, pragmatic and acceptable model of care is likely to result.
- Funding has limited the scope of the study to two sites, with a focus on one group of immigrant women.

INTRODUCTION

Sweden has a clearly stated goal of providing equitable health care for the whole population, which in recent decades has become increasingly diverse. Despite this, studies indicate that pregnancy outcomes among immigrant women are suboptimal compared with those of Swedish-born women(1). These health disparities point to the need for implementing and evaluating interventions to improve care for immigrant women and their families. This study protocol describes the development of an intervention to improve antenatal care (ANC) for Somali-born women and families giving birth in Sweden.

Somali-born women constitute one of the largest groups of immigrant women of childbearing age in Sweden(2), after more than two decades of political instability in Somalia. Of all immigrant women, they are known to be at high risk of maternal and perinatal morbidity and mortality both in Sweden(3-5) and internationally(6). The reasons are complex and include both pre- and post-migration factors. Insufficient health care provision and low socioeconomic conditions in the country of origin influence women's health status (7). After migration to Sweden, later and lower attendance for antenatal care (8), and poorer experiences of maternity care(9) have been reported. Language difficulties and lack of familiarity with care systems, and discrimination and sub-optimal care are contributing factors(3, 10,11). Furthermore, higher rates of anaemia, insufficient weight gain and infants born small for gestational age(8) indicate that Somali women are not sufficiently

For peer review only - http://bmjopen.bmj.com/site/about/guidelines.xhtml

reached by advice about prevention or treatment. Lower attendance at childbirth and parent education classes among immigrant women has also been found, with classes most often held in Swedish(12).

Few measures have been taken in Sweden to reduce barriers to maternity care for immigrant women or to better meet their care needs. Furthermore, few studies have investigated how care might be designed in ways that are both attractive and acceptable to immigrant women and to midwives who are the main providers of care during pregnancy and childbirth. Respect, communication, community knowledge and care tailored to women's circumstances and needs have been proposed as central components in quality maternity care(13). In line with this, a systematic review in five countries highlighted that immigrant women want the same things from care as non-immigrant women: care should be of high quality and safe, with adequate information and support and attentive to individual needs. The review also found however, that immigrant women consistently reported experiencing additional challenges, including communication difficulties, lack of familiarity with health care systems and discrimination(10).

Language barriers, marginalisation and prevailing cultural stereotypes pose challenges in designing, implementing and evaluating innovative care models including, rather than excluding, immigrants. These factors need to be carefully addressed before widespread change of new models of care are introduced. Participatory approaches have shown promising results(14-16) and reduce the risk of enhancing marginalisation. Focus on communication and meaningful dialogue throughout intervention design, recruitment, data collection, implementation and evaluation is valuable (15).

In Sweden, the care of pregnant women during normal pregnancies is provided at community-based ANC clinics by registered midwives, based on national and regional guidelines(17). Included are 8-10 pregnancy check-ups free of charge, traditionally offered through individual appointments (30 minutes) with the same midwife throughout the pregnancy. Extra visits are booked and specialised medical referrals are made when needed. Parent education including preparation for childbirth and parenting is provided during the individual appointments or in groups/classes, mainly in Swedish and often focusing on first time parents. Partners are encouraged to participate.

One innovative approach for improving antenatal care is group antenatal care (gANC). This care model incorporates pregnancy assessments and parent education in small groups, either with shorter individual appointments with the midwife at the beginning or end of the group session, or with

For peer review only - http://bmjopen.bmj.com/site/about/guidelines.xhtml

individual clinical checks by the midwife provided in the group space (18-20), while the rest of the group can socialise. Self-assessments of blood pressure and urine have also been included in some gANC models(18, 20). Results from studies in the US, Iran and Sweden report improved satisfaction with care(19, 21-24), improved iron taking, higher birthweight, lower caesarean section rates, more breastfeeding and earlier diagnosis of complications(23). Higher self-esteem and lower levels of stress, social conflicts and depression have been reported, as have more visits to ANC by fathers-to-be, increased support in social contacts with other parents, and reduced costs(18, 19, 23, 25-28), despite more time spent with the midwife throughout pregnancy. gANC thus appears to enhance women's agency, dialogue, time with the midwife and social interaction, and to improve women's views of their care and also some of their pregnancy outcomes. gANC may be especially valuable for specific groups of women, as seen for example in studies among young African-American women in the United States(26, 29) and among Karen refugee women in Australia(15).

The *Hooyo-project* ('mother' in Somali) is a gANC initiative developed in Sweden in response to the evidence about lack of equity in pregnancy care provision and poorer outcomes for Somali-born women. gANC has not been evaluated among immigrant women and families in Sweden, yet it may well be a model of care that can break down cultural and care barriers, enable better dialogue and understanding between caregivers and immigrant women and improve women's satisfaction with their care, especially if language support is provided(15).

The overall aim for this study is to develop and test the acceptability, and immediate impacts of gANC for Somali women residing in Sweden, in an effort to improve experiences of antenatal care, knowledge about childbearing and the Swedish health care system, emotional wellbeing and ultimately, pregnancy outcomes.

Specific objectives include

1) To establish with Somali-born women and antenatal caregivers the acceptability and feasibility of gANC and to develop an appropriate model in order to improve outcomes (Phases 1 and 2);

2) To establish appropriate recruitment and data collection procedures and outcome measures in consultation with the Somali community and with care providers in order to evaluate the new model (Phase 2) ; and

3) To implement and evaluate an agreed model of gANC in partnership with the Somali community and antenatal caregivers (Phases 3 and 4).

For peer review only - http://bmjopen.bmj.com/site/about/guidelines.xhtml

For peer review only

METHODS AND ANALYSIS

Design

An intervention development and feasibility study including focus group discussions, a historically controlled evaluation and process evaluation(30, 31). The intervention is implemented in two antenatal care clinics in mid-Sweden. The feasibility and piloting includes testing procedures for acceptability, estimating the likely rates of recruitment and retention of women, and the calculation of appropriate sample sizes for future controlled trials. Complex interventions that are adjusted to local, specific(32) or personal needs(33) are likely to be more effective than completely standardised models(30). Key principles underpinning the study are therefore:

- Active involvement of Somali parents and midwives in needs assessment and care design
- Attention to language and contextual factors
- Flexibility in study methods to respond to issues as they arise
- A care model ready to continue or be replicated after the project concludes with only minor adjustments.

Hooyo consists of four phases with process evaluation activities informing study progress. *Phase 1* is the preparation phase, including needs assessment, the development of contextual understanding and building a logic model for the project. *Phase 2* includes the development of the intervention and of the evaluation tools and *Phase 3* involves implementation and evaluation of the intervention using historical controls. In *Phase 4* the implementation process, the feasibility and the mechanisms of impact are evaluated, including contextual factors, using Moore's Process Evaluation of Complex Interventions as an overarching framework(31). Phases 1 and 2 were conducted in 2016-2017. Recruitment and data collection for the historically controlled evaluation (Phase 3) and process evaluation (phase 4) commenced in late 2016 and will be completed during 2020. Women in the control group were recruited between October 2016 and April 2018 and recruitment of women into the intervention began in May 2018 and will be complete in December 2019. Final data collection will be completed by July 2020.

For peer review only - http://bmjopen.bmj.com/site/about/guidelines.xhtml

Phase 1 – Preparation phase

The purpose of Phase 1 was to understand how current individual care was delivered and experienced and what changes women, partners and midwives thought might improve care, taking into account contextual factors that might have an impact on the development, structure and content of a new model. The aim was also to assess whether a language-supported group antenatal care model might be appropriate and acceptable to address concerns raised about current care.

Initial dialogue and choosing study settings

In the municipality chosen for *site one,* the number of Somali immigrants had increased fivefold during the last decade and the ANC midwives had begun to re-think how they were providing care. At the same time, there was awareness of lower ANC attendance and adverse outcomes among child-bearing Somali-born women in Sweden(8), and a need for more appropriate explanations of ANC routines had been highlighted in interviews with Somali-born women(34). This prompted a dialogue between the principal investigator and the ANC midwives, outlining the possibility of evaluating an alternative model of care. The ANC clinic is located in a Primary Health Care Centre staffed by 10 midwives, within a public hospital located outside the city centre. It caters for approximately 75% of all pregnant women in the municipality and almost all of the pregnant immigrant women in the municipality, which in total has 50,000 residents. The majority of the Somali-born women visiting the clinic live in two areas situated approximately five kilometres away, necessitating use of a car or public transport.

At *site two*, the ANC clinic is located in a Family Health Centre in a suburb of the capital city. Close collaboration takes place between ANC midwives, social workers, child health nurses and the open playgroup located in the same building. Three ANC midwives are employed and the target area includes two residential areas with different socio-demographics; with primarily Swedish-born residents in one area and non-Swedish born residents and second generation immigrants in the other. Somali-born residents have lived in this area for more than 25 years and midwives reported that most Somali-born pregnant women and families were able to communicate in Swedish. Integration of families of different cultural backgrounds is actively encouraged at the clinic. Initial discussions revealed that the midwives believed group antenatal care for women of mixed cultural backgrounds (with interpreters available if needed) would contribute positively to integration. As a result, it was agreed that integrated groups would be more appropriate and acceptable at this site.

For peer review only - http://bmjopen.bmj.com/site/about/guidelines.xhtml

Mechanisms for engagement: Reference group and bicultural research assistants

A project reference group was established comprising research team members, antenatal care midwives and representatives of the Somali community from both sites. Terms of reference were developed, including provision of advice to the research team on design of the intervention, appropriate recruitment processes and data collection methods; development of study questionnaires; networking with Somali associations and ANC clinics; as well as contributing to the interpretation and dissemination of findings throughout the project period.

A bilingual Somali research assistant with a health care background was employed full time in the project at site one. This enabled networking with Somali community members, bridging language gaps, input into questionnaire design, recruitment of participants, data collection and arranging and interpreting focus group discussions together with research team members. At site two, focus group discussions were facilitated by two Somali community workers, including recruitment and interpreting.

Participatory focus group discussions with Somali parents and antenatal care midwives

To develop understanding of current antenatal care and how it was experienced, Phase 1 included focus group discussions (FGDs) with Somali-born parents and with ANC midwives; presented in brief below and in Ahrne et al(35).

Parents were recruited using purposeful sampling. In total, 16 mothers and 13 fathers with varied length of stay in Sweden and recent experience (<2 years) of ANC were included. Seven ANC midwives were recruited purposefully from both sites and from an additional site where the midwives had previous experience of group antenatal care with Somali-born parents.

Three focus group discussions were held with Somali-born mothers, two with Somali-born fathers and three with ANC midwives. Emerging themes were highlighted by the researchers and crosschecked during the discussions with participants for accuracy(36, 37). Thematic analysis as described by Attride-Sterling(38) was used to interpret data from the FGDs.

Key findings informing intervention development

Challenges at the system level and in the care encounter in striving for optimal ANC were identified(35); alongside findings that could directly inform the intervention development. Instead of focusing on specific cultural or ethnic aspects of care, the results indicated a need to maintain focus on diversity and individual needs. The need for forums for social interaction, and for dialogue

For peer review only - http://bmjopen.bmj.com/site/about/guidelines.xhtml

which could bridge gaps between midwives' and women's understanding of the purpose of antenatal care, improve communication and reduce the risks for stereotyping was apparent. Responding to a wide range of health literacy needs, welcoming fathers and safeguarding an open atmosphere were other issues raised. Furthermore, practical issues such as optimal location and times for ANC visits were discussed. The findings contributed valuable input to the program theory and to the decisions regarding underpinning central principles for the coming intervention (Box 1), as well as for the development of the structure and content of the intervention in Phase II.

For peer review only

For peer review only - http://bmjopen.bmj.com/site/about/guidelines.xhtml

Problem statement	Conceptual framework and rationale	Hooyo: A group approach to improving ANC	Hypothesised mechanisms of effect	Desired outcomes
**Current ANC in Sweden may not provide equitable care for Somali-born women:**<br>• Lower participation in antenatal care<br>• Poorer birth outcomes<br>• Communication difficulties<br>• Lack of familiarity with Swedish antenatal care structures<br>• Lower attendance in parental education<br>• Negative attitudes and suboptimal care<br>**Initial FGDs with Somali-born parents/ANC midwives highlight need for:**<br>• Improved communication and dialogue<br>• Bridging gaps between divergent health literacy knowledge<br>• Care free from generalisa-tions, tailored to individual needs<br>• Clearly described expecta-tions regarding partner's role	**Core values for quality care:** respect, communication, community knowledge and understanding<br><br>**Person-centred care** to identify and address women's individual needs<br>**Continuity of care** for positive care experiences and health outcomes<br><br>**Group antenatal care** a promising alternative to individual visits:<br>• More positive views of care<br>• Some positive impacts on birth outcomes<br>• More time with midwives and more comprehensive parental education<br>• In Sweden studied with Swedish-speaking groups only<br><br>**Key underpinning principles**:<br>• Active involvement of Somali parents/ midwives in assessment and care design<br>• Attention to language and contextual factors<br>• Flexibility in study methods to respond to issues as they arise<br>• A care model ready to continue or replicate after project ending with minor adjustments	**Language supported group antenatal care,** involving<br>• 8-9 group sessions 1 1/2 hours with 6-8 women (partners welcome) from gest. week 24<br>• Facilitated by two midwives assisted by interpreter<br>• Brief individual midwife check-ups incorporated<br>• Childbirth/parenting themes with focus on dialogue and discussion<br>• Motivational interviewing for groups as a vehicle for focusing care on women's needs<br>• Adjustments based on local needs at each site:<br>*Site 1:* Groups specifically for Somali-born<br>*Site 2*: Groups with diverse backgrounds and languages	**Interpreter-supported group dialogue facilitated by midwives will result in**<br>• Improved communication →better suited care<br>• More time for discussions →mutual understandings in views around childbirth and health promotion → strategies for improving outcomes<br>• An additional arena for social contact and support →increased well-being<br>• Combining pregnancy check-ups with groups →motivation for attending ANC, and parental education<br>• Common language/ background → understanding and empower women to raise voices in having needs addressed<br>• Mixed groups →integration and understanding through cross-language/culture interactions	**Women:**<br>• Happier with the ANC<br>• More confident in and knowledgeable about the pregnancies<br>• Improved wellbeing<br>• Improved attendance at antenatal care visits<br>• Improved uptake of health advice<br>• Ultimately; improved pregnancy outcomes<br><br>**Partners:**<br>• Feeling welcomed and included<br>• Increased understanding of expectations<br><br>**Midwives**:<br>• Improved understanding of womens needs<br>• Feel better able to share health knowledge in meaningful ways<br>• Provide more supportive, non-judgemental care<br>• Positive about benefits of group care

Box 1. Logic model of Hooyo including problem statements, conceptual framework and rationale, the Hooyo group antenatal care model, hypothesised mechanisms of effect and desired outcomes.

For peer review only - http://bmjopen.bmj.com/site/about/guidelines.xhtml

Phase 2 – Development of the intervention and study evaluation tools

Developing group antenatal care

Prior to the study commencing, a review of the literature on migrant women's birth outcomes, experiences of antenatal care and on alternative models of care for migrant women had been undertaken, including group antenatal care. This, together with the experiences of team member EA in evaluating group antenatal care in Sweden(19, 24, 28), led us to investigate whether group antenatal care could be appropriately developed to improve outcomes for Somali-born women. The preliminary findings from the FGDs confirmed language supported gANC as a possible alternative care model and this was discussed in the Reference Group. Research team members participated in discussions at each study site during these processes. The intervention development was characterised by constant modifications to fit local prerequisites at both sites, including local guidelines and routines in the ANC clinics, and needs among the women; not all of which were apparent from the start.

Underpinning principles

Central findings in the FGDs were the desire for individualised care, a need for forums for dialogue to address variations in health literacy and health care understanding, and at the same time respond to the request to move beyond stereotypes based on ethnicity or "culture". This led us to focus on Person Centred Care (PCC)(39) as a foundation for the intervention, and on how this could be strengthened and encouraged through active dialogue in group-based care. This was also in line with the ongoing implementation of PCC generally in the health system in Sweden. In searching for a method to support this approach, the midwives proposed Motivational Interviewing (MI). They had already received some training in MI for use in individual appointments, and now MI *for groups* was added. Principles of person-centred care and MI encourage understanding the person as an individual, and developing partnership and promoting self-efficacy for which an active and open dialogue is central(39-41).

Addressing language and integration

To respond to the diverse needs identified at the two ANC clinics and to assess advantages and disadvantages with both homogeneous and heterogeneous groups regarding language and cultural background, it was decided that gANC would be offered to Somali-born women at site 1. A large number of Somali migrants had settled in this municipality in recent years and this meant that many were not yet fluent in Swedish. At site 2, gANC would be offered to *all* women attending the clinic,

For peer review only - http://bmjopen.bmj.com/site/about/guidelines.xhtml

in integrated groups. At site 1, the need for a Somali interpreter at each session was anticipated. Since a finding in the FGDs was that continuity of known Somali interpreters had been a previous success factor, it was decided to engage two experienced interpreters who could alternate. At site 2 it was anticipated that the need for interpreters in different languages would vary and thus they would be engaged when needed.

The intervention: Group Antenatal Care (gANC)

The intervention consists of gANC offered at both sites, modified to the needs in each site and group. Women are allocated to a group with women at similar stages of pregnancy within a four-week gestational age span. From the second visit (gestational week 20-26) they receive group antenatal care. Each group will consist of 6-8 women and partners (or another support person) for optimal group dynamics and dialogue. While reports of gANC internationally involve numbers in groups of 5-20 in low to middle income countries (42) and 8-12 in high income countries (43), the need to provide interpreting during groups sessions led to the choice of a somewhat smaller group size as being appropriate to allow all group members a chance to participate in the discussions. Partners are generally encouraged to participate in Swedish ANC, and hence this will be the point of departure also for the gANC, but with the final decision about partner participation taken by the women included in each group. Frequency and number of sessions follow the national Swedish program for antenatal care, i.e. 8-9 appointments during a normal pregnancy. Each visit includes a group session for one hour, facilitated by one of two midwives assigned to the group, and with interpreter assistance. Although each group session has a focus on a theme related to pregnancy, birth or parenting in line with national recommendations(17), particular emphasis will be given to issues and questions raised by the participants. Group care allows women to talk with each other and share their experiences in their own language. They also hear midwives' responses to other women's questions, expanding the dialogue and information sharing that can occur between midwives and women. The presence of an interpreter facilitates communication between the women and the midwives.

Alongside the group discussion each woman also has a 15-minute individual appointment with the midwife responsible for her care, who is the same midwife for each appointment according to Swedish National Guidelines(17).   Routine pregnancy controls are carried out and the woman can also raise any particular issues with her midwife during this time. In total the time for each visit will be approximately 75 minutes instead of 30 minutes, as is common for standard individual care (though this does not take into account the time midwives also spend providing childbirth and

For peer review only - http://bmjopen.bmj.com/site/about/guidelines.xhtml

parenting classes). Additional individual appointments can be booked as needed if medical or other issues arise.This model for group antenatal care draws on the model used in a previous Swedish study by Andersson et al(19, 24, 28), but differs in that two midwives and a language interpreter are present throughout the group sessions and the principles of PCC and MI are explicitly identified as the theoretical underpinnings for care in the group.

Development of Evaluation Tools

Questionnaires

Questionnaires for data collection from participating women were developed by the research team. A number of questions were taken from the Migrant Friendly Maternity Care Questionnaire (MFMCQ)(44), in some cases slightly modified to fit the purpose of our study, as is the intention in use of the MFMCQ, and for use both in pregnancy and after birth, including occasional changes of tense from the original. Selected questions were also included from the Cambridge Worry Scale (11 single-item questions on worries about the upcoming labour and birth), which is available in English and Swedish(45), and the ten-item Edinburgh Postnatal Depression Scale (EPDS) which is available in English, Swedish and Somali(46). The questionnaires were developed in English by the research team and translated to Swedish by one of the bilingual team members and then cross-checked by another bilingual researcher in the team. Thereafter the questionnaire was translated into Somali by a professional Somali translator, fluent in English, Swedish and Somali, and finally cross-checked by a Somali-speaking research assistant. At each step of the process the questionnaire was pilot-tested with Somali-born women for relevance and understanding with adjustments made accordingly. During piloting it was clear that some of the items and response alternatives in the EPDS were difficult for women. Although the scale is translated into Somali, no validation study has to date been published. As data are collected in face-to-face or telephone interviews conducted by the bilingual research assistant, a decision was made to include the EPDS as translated, with the research assistant able to explain the meaning of the questions in plain language if needed. Alongside the current study, we have commenced a process for validating the EPDS Somali translation, involving think-aloud interviews(47) with Somali-born women, in order to contribute to evidence about use of the EPDS in Somali.

Outcome measures

Primary outcomes are women's overall ratings of antenatal care and emotional wellbeing. Ratings of care are assessed by the core question “When thinking about your overall experience of antenatal

For peer review only - http://bmjopen.bmj.com/site/about/guidelines.xhtml

care - in general, have you been happy with the care that you have received?" with response alternatives *always, mostly, sometimes, rarely and never*. The question is modified from the Migrant Friendly Maternity Care Questionnaire (MFMCQ)(44) and more detailed questions about specific aspects of care are also included. Emotional wellbeing is assessed with the Edinburgh Postnatal Depression Scale (EPDS), completed in English, Swedish or Somali as appropriate (46).

Secondary outcomes are adequate number of visits measured by the Adequacy of Prenatal Care Utilization Index (APCUI), i.e. expected number of visits in relation to actual number adjusted for gestational week at booking visit and delivery(48), social support during pregnancy using slightly modified questions from the Pregnancy Risk Assessment Monitoring System (PRAMS)(49) and worries about the upcoming birth using questions from the Cambridge Worry Scale(45). Knowledge about danger signs and where to seek health care if particular symptoms arise are assessed by questions with multiple choice responses, such as "Now at the end of your pregnancy, where would you turn if you had bleeding from the vagina? When would you seek care?". From the patient record, the following are retrieved: estimated time spent with the antenatal care midwife, gestational age at the first ANC visit, lowest measurements of Haemoglobin and S-Ferritin, weight gain, attendance at parent education, mode of birth, interventions (induction of labour, pain relief including epidural, oxytocin), blood loss, diagnoses (mother) and gestational age, birth weight, Apgar score, umbilical cord pH, and the need for neonatal intensive care (infant).

Preparing for implementation

Awareness raising

To create awareness and interest in the project and subsequently in the new model of care, Somali and other community networks are involved in different ways. Information is disseminated by participants in the reference group, by researchers and research assistants one-to-one, and in small groups gathering Somali-born women. A brief pamphlet in Swedish and Somali has been produced and distributed among women, Somali community associations, open playgroups and activity centres and as a poster in the waiting rooms at participating ANC clinics.

Training of midwives and interpreters

Areas to be covered in a preparatory training workshop for midwives were identified through findings in the FGDs, in dialogue with the midwives at both sites and in reference group meetings. The research team then tailored a 1 ½ day workshop for all midwives at both sites, held prior to intervention commencement. In response to the needs expressed, the main focus was group

For peer review only - http://bmjopen.bmj.com/site/about/guidelines.xhtml

processes including person-centred care(38) and motivational interviewing (MI) in groups(40). A two-day follow-up session is planned during the intervention period to enable feedback on implementation and address any issues arising.

The interpreters' preparation included two hours of information regarding the structure of the ANC care system, introduction to the Hooyo-project and the gANC model emphasising dialogue and MI-principles, participation in meetings with involved midwives and receiving a written project manual (described below).

Development of material for midwives leading group sessions

A manual comprising information and suggestions for the content and structure of group sessions has been developed, which the midwives are free to use and adjust according to the needs in the groups. An overview of the availability of evidence-based resources related to pregnancy and birth led us to recommend the use of existing resources where available, as they are free of charge and often in different languages. This also adds to the sustainability and cost-effectiveness of the intervention, and makes it easier to duplicate. A study-specific homepage was created, comprising the same information and support as in the written manual.

Phase 3 – Design and methods for the historically controlled evaluation of the intervention

Participants and recruitment

At both sites, primiparous and multiparous Somali-born women are recruited to the study. Recruitment and retention of participants to clinical studies is a well-documented challenge in research across languages and cultures(50). We therefore thoroughly planned how to optimise the recruitment and information processes in relation to eligible women and adjusted processes to local prerequisites at both sites. The Somali-speaking research assistant is based at site 1 where most needed, and where the majority of the recruitment is performed. In site 2, team-member MA is involved with recruitment, information and data collection, supported by the Somali-speaking research assistant form site 1. All pregnant Somali-born women attending the two ANC clinics receive brief information about the study from the midwives during their first visit and if they agree to receive further information, they are then contacted by the research assistant or MA after the first ANC visit, either by telephone or face to face for further oral and written information in Somali, English or Swedish, as needed.

For peer review only - http://bmjopen.bmj.com/site/about/guidelines.xhtml

For peer review only

Inclusion criteria are being born in Somalia and <25 weeks of gestation. *Exclusion criterion* is a health condition preventing participation in group antenatal care, i.e. severe mental health condition or if the pregnancy care were to be transferred to a specialist obstetric clinic. During the first 18 months of the study, recruited women were included in the control group receiving standard individual care and in the following 18 months women are included in the intervention group. All women are informed verbally and orally about the purpose of the study and that participation is voluntary. Women included in the control group were informed that their perspectives about their care would contribute knowledge valuable for a proposed intervention study aimed at improving antenatal care for migrant women.

Data collection for the intervention

Questionnaires

Questionnaire data are collected at three time points (Q1, Q2, Q3). The baseline measurement (Q1) is conducted after recruitment, i.e. before gestational week 25. Women are contacted for a face-to-face or telephone structured interview in Somali, Swedish or English at a place of their choice. Q1 includes questions on background (parity, education, income, occupation), migration (time of residence, reason for migration, migration status, language) and social support, psychological and physical wellbeing, knowledge of danger signs and the health system, and expectations about the up-coming birth. Data for Q2 are collected in late pregnancy, after gestational week 35, in the same manner. Q2 includes questions on experiences of care and information during the ongoing pregnancy, knowledge of the health system, pregnancy and birth, social support, and psychological and physical wellbeing and expectations for the upcoming birth. Q3 is administered two months postpartum and includes questions regarding the overall experience of pregnancy, ANC, birth and psychological and physical wellbeing. After each interview, a small gift is offered as a token of appreciation for women's participation.

Medical records

Data for secondary outcome measurement will be collected from patient medical records, for all consenting participants. This includes data related to pregnancy, labour and birth and maternal and infant outcomes.

For peer review only - http://bmjopen.bmj.com/site/about/guidelines.xhtml

Sample size issues

Our main purpose is to develop and test the feasibility of the intervention and study processes and in so doing, also provide information to inform sample size calculations for possible future randomised controlled trials. Nevertheless, based on available birth rate data we estimate being able to include 100 women in the control group and 100 women in the intervention group during the study period. We estimate that the study will have the power to detect a clinically relevant increase in women's overall satisfaction with antenatal care from an expected 65% among Somali women receiving individual care to 82% in those receiving gANC (approximating rates for Swedish speaking women in a national population based study(51) with 80% power and an alpha of 20%, with 70 women in each group. To have similar power to detect differences in mean scores on the Edinburgh Postnatal Depression Scale (a hypothesised reduction from a mean of 8.0 in the control group – similar to that found in studies of migrant women, to 6.0 in the gANC group – similar to that found in Swedish population-based studies(52), 63 women are required in each group. Allowing for 20% loss to follow-up at the time of data collection with women two months postpartum, 174 women will need to be recruited.

Differences between groups will be described as odds ratios and 95% confidence intervals after estimation by means of logistic regression analyses. Comparison of means will be undertaken using t-tests where data are normally distributed, or medians compared using Mann-Whitney U tests if not.

Phase 4 –Design of the process evaluation

A process evaluation using a mixed methods approach will be conducted throughout the study (30). The process evaluation will address whether the intervention was carried out according to plan, what was achieved, if and what adaptations were needed, who was reached by the intervention, any unexpected events and will also aim to understand the mechanisms of impact and what mediated these mechanisms. The process evaluation will also address contextual factors(31).

Process evaluation data collection and measures

Data collection for the process evaluation(31) will be nested in the different phases, supported by different measurement tools and activities. Moore's process evaluation framework(31) will guide the data collection, analysis and interpretation. The framework emphasises the impact of, and relationship between the intervention, implementation, mechanisms and context, and a topic guide and analysis will focus on *what* is achieved and *how* (fidelity, dose, adaptations, reach). Focus for

For peer review only - http://bmjopen.bmj.com/site/about/guidelines.xhtml

the mechanisms of impact will be the participants' interactions with the intervention and potential mediating factors.

A session checklist for completion by the midwife after each group session will cover topics discussed, resources used, number of participating women and support people, group dialogue and the midwife's brief view of each session. The checklist was developed by the research team, discussed with the reference group and, after adjustments, presented to all included midwives for further modifications, which were addressed by the researchers before intervention start.

Participant observations during the group sessions will contribute data describing the delivery of gANC and the mechanisms of impact by describing group dynamics, dialogue and active participation by women in group sessions. A protocol for observations during group sessions developed by the research team, focuses on group mechanisms, dialogue and interaction, based on PPC and MI principles. This will provide the guide for participant observations by one or two members of the research team during a number of randomly selected group sessions.

Participant experiences of group antenatal care. Questionnaire data for all participating women and qualitative data collected from women, midwives, partners, interpreters and heads of departments will describe responses to and interactions with the intervention and their views regarding the feasibility of the intervention and mechanisms of impact. Women's experiences of the content, structure and feasibility of gANC will be assessed in the *follow-up questionnaire* (Q3) at 2 months postpartum. Additionally, the questionnaires will identify a sample of women with a diversity of experiences to be recruited for *more in-depth individual interviews*. Midwives' experiences of the same, and their perceptions regarding mechanisms of impact will be collected through *qualitative FGDs or interviews* as appropriate. Partners' and interpreters' views will be collected through *qualitative individual interviews* upon completion of participation. FGD and interview data will be collected using a topic guide with open-ended questions developed by the research team and analysed using deductive content analysis according to Elo and Kyngas(53). *Field notes* taken by the researchers in dialogue with research assistants, involved midwives and heads of departments will assist in describing the choices and decisions made during the implementation process.

Patient and public involvement

The initial literature review informing the research questions included studies focusing on immigrant women's experiences and views of maternity care post-migration, including the views of Somali-born women. Initial focus group discussions for the study included Somali women and men

For peer review only - http://bmjopen.bmj.com/site/about/guidelines.xhtml

with recent experiences of antenatal care and these informed the development of the intervention. As described above, a study reference group involves Somali community members in all stages of the project and a bicultural research assistant is involved in recruiting women to the study. All participants will receive a summary of the results in their preferred language and seminars will be held with stakeholder groups.

DISCUSSION

The Hooyo study is an attempt to address some of the health and care disparities in the Swedish antenatal care system. The development and feasibility design of the study(30) together with the nested process evaluation(31) will contribute valuable information for future randomised controlled studies, as well as in the design of antenatal care interventions focused on reducing inequalities in health between immigrants and non-immigrants. The study will provide guidance on the acceptability of this model of care among Somali-born women, their partners and midwives in Sweden, and on recruitment and data collection from responders who are often excluded from research due to communication barriers or marginalisation. The study will also indicate whether gANC has a positive impact on Somali women's experiences of antenatal care and might therefore be appropriate for other migrant groups.

For the midwives, the intervention provides a platform for reflection and dialogue about improving care for migrant women. This is vital, since care provision is always an interplay between care-givers and care-receivers. The early inclusion of midwives' and participants' views provided space to shape the planned intervention in relevant ways in terms of content, structure and underlying core principles(33). By emphasising dialogue, a person-centred approach and the active participation of women, partners and midwives throughout development and implementation of the intervention, we hope to develop a relevant, pragmatic and acceptable model of care for this group of immigrant women, and one that might replicate well to other settings and groups with minor adjustments. The challenge is to develop and implement an acceptable model of care addressing diverse needs of the care-receiving group and suited to local conditions, while ensuring that medical and public health guidelines are fulfilled. The findings of the study will determine if this can be achieved.

It could be argued that the study should include more antenatal care sites and a range of different immigrant groups. Funding limitations meant restricting the study to two sites and focusing on only one immigrant group, something that may limit the study's generalisability. On the other hand, it is

For peer review only - http://bmjopen.bmj.com/site/about/guidelines.xhtml

expected that many of the strategies employed to develop and test group antenatal care in this study, and the lessons learned in doing so, will have future relevance in improving antenatal care for immigrant women more generally.

ETHICS AND DISSEMINATION

The study is approved by the Regional Ethical Review Board, Stockholm, Sweden, 2015-12-04, Dnr 2015/1703-31/1. All participants are offered information about the study, informed about the voluntary nature of participation in detail and give their written consent prior to enrolment.

The findings of the study will be disseminated at relevant national and international conferences and through publications in peer-reviewed journals. Seminars involving local stakeholders in the communities, county council representatives and practitioners will further provide a platform for dissemination and reflections about lessons learned at the end of the project. The findings of the study will also be presented via the Hooyo-project website https://ki.se/kbh/modrahalsovard-for-utlandsfodda-kvinnor-hooyo-projektet.

DECLARATIONS

Acknowledgements
The authors would like to thank the midwives, members of the Hooyo reference group and all the Somali-born women and men who have contributed in the preparation phase of the study.

Consent for publication

Not applicable.

Availability of data and material
The dataset generated and analysed during the preparation phase of the current study is not publicly available due to restrictions based on Swedish law safeguarding individual privacy. It may be made available upon reasonable request.

Competing interests

The authors declare that they have no competing interests.

Funding

This study has received funding from the Swedish Research Council [grant number 2015-02470], Forte [grant number 2016-00957] and the Doctoral School in Health Care Sciences, Karolinska

For peer review only - http://bmjopen.bmj.com/site/about/guidelines.xhtml

Institutet [grant number 2-144/2016]. The funding bodies have played no role in the design of the study, nor in data collection, analysis and interpretation of the data or in writing the manuscript.

Author statement

ES, RS, EA and BE initiated the study. UB, ES, RS, EA MA, BE, AA, FH, KT, YL, MI and AB contributed to planning and design. UB, MA, ES, RS, EA AA and FH developed questionnaires and topic guides. UB drafted the manuscript, MA, ES, RS, UB, EA, BE, AA, FH, KT, YL, MI and AB revised it. All authors read and approved the final manuscript.

REFERENCES

1 National Board of Health and Welfare. Socioekonomiska faktorers påverkan på kvinnors och barns hälsa efter förlossning. Stockholm: National Board of Health and Welfare; 2016.

2 Statistics Sweden [Internet]. [Accessed 2018 Aug 20]. Available from: http://www.statistikdatabasen.scb.se/.

3 Esscher A, Binder-Finnema P, Bodker B, et al. Suboptimal care and maternal mortality among foreign-born women in Sweden: maternal death audit with application of the 'migration three delays' model. *BMC Pregnancy Childbirth* 2014;14:141.

4 Essen B, Bodker B, Sjoberg NO, et al. Are some perinatal deaths in immigrant groups linked to suboptimal perinatal care services? *BJOG* 2002;109(6):677-82.

5 Essen B, Hanson BS, Ostergren PO, et al. Increased perinatal mortality among sub-Saharan immigrants in a city-population in Sweden. *Acta Obstet Gynecol Scand* 2000;79(9):737-43.

6 Small R, Gagnon A, Gissler M, et al. Somali women and their pregnancy outcomes postmigration: data from six receiving countries. *BJOG* 2008;115(13):1630-40.

7. Ali AM, Handuleh J, Patel P, et al. The most fragile state: healthcare in Somalia. *Med Confl Surviv*. 2014;30(1):28-36.

8 Rassjo EB, Byrskog U, Samir R, et al. Somali women's use of maternity health services and the outcome of their pregnancies: a descriptive study comparing Somali immigrants with native-born Swedish women. *Sex Reprod Healthc* 2013;4(3):99-106.

9 Berggren V, Bergstrom S, Edberg AK. Being different and vulnerable: experiences of immigrant African women who have been circumcised and sought maternity care in Sweden. *J Transcult Nurs* 2006;17(1):50-7.

For peer review only - http://bmjopen.bmj.com/site/about/guidelines.xhtml

10 Small R, Roth C, Raval M, et al. Immigrant and non-immigrant women's experiences of maternity care: a systematic and comparative review of studies in five countries. *BMC Pregnancy Childbirth* 2014;14:152.

11 Binder P, Borne Y, Johnsdotter S, et al. Shared Language Is Essential: Communication in a Multiethnic Obstetric Care Setting. *J Healthc Commun* 2012;17(10):1171-86.

12 Fabian H, Radestad I, Rodriguez A, et al. Women with non-Swedish speaking background and their children: a longitudinal study of uptake of care and maternal and child health. *Acta paediatrica* 2008;97(12):1721-8.

13 Renfrew MJ, McFadden A, Bastos MH, et al. Midwifery and quality care: findings from a new evidence-informed framework for maternal and newborn care. *Lancet* 2014;384(9948):1129-45.

14 Osman F, Salari R, Klingberg-Allvin M, Schon UK, et al. Effects of a culturally tailored parenting support programme in Somali-born parents' mental health and sense of competence in parenting: a randomised controlled trial. *BMJ open* 2017;7(12):e017600.

15 Riggs E, Muyeen S, Brown S, , et al. Cultural safety and belonging for refugee background women attending group pregnancy care: An Australian qualitative study. *Birth* 2017;44(2):145-52.

16 Riggs E, Yelland J, Szwarc J, et al. Promoting the inclusion of Afghan women and men in research: reflections from research and community partners involved in implementing a 'proof of concept' project. *Int J Equity Health* 2015;14:13.

17 The Swedish Society of Obstetrics and Gynecology (SFOG) and The Swedish Association of Midwives. Mödrahälsovård, Sexuell och Reproduktiv Hälsa. 2016; ARG report 76.

18 Lathrop B. A systematic review comparing group prenatal care to traditional prenatal care. *Nurs Womens Health* 2013;17(2):118-30.

19 Andersson E, Christensson K, Hildingsson I. Mothers' satisfaction with group antenatal care versus individual antenatal care - a clinical trial. *Sex Reprod Healthc* 2013;4(3):113-20.

20 Hunter L, Da Motta G, McCourt C, et al. `It makes sense and it works': Maternity care providers' perspectives on the feasibility of a group antenatal care model (Pregnancy Circles). *Midwifery* 2018;66:56-63.

21 Ickovics JR, Kershaw TS, Westdahl C, et al. Group prenatal care and perinatal outcomes: a randomized controlled trial. *Obstet Gynecol* 2007;110(2 Pt 1):330-9.

22 Jafari F, Eftekhar H, Mohammad K, et al. Does Group Prenatal Care Affect Satisfaction And Prenatal Care Utilization in Iranian Pregnant Women? *Iran J Public Health* 2010;39(2):52-62.

For peer review only

For peer review only - http://bmjopen.bmj.com/site/about/guidelines.xhtml

23 Jafari F, Eftekhar H, Fotouhi A, Mohammad K, Hantoushzadeh S. Comparison of maternal and neonatal outcomes of group versus individual prenatal care: a new experience in Iran. *Health Care Women Int* 2010;31(7):571-84.

24 Andersson E, Christensson K, Hildingsson I. Parents' experiences and perceptions of group-based antenatal care in four clinics in Sweden. *Midwifery* 2012;28(4):502-8.

25 Wedin K, Molin J, Crang Svalenius EL. Group antenatal care: new pedagogic method for antenatal care--a pilot study. *Midwifery* 2010;26(4):389-93.

26 Ickovics JR, Kershaw TS, Westdahl C, et al. Group prenatal care and perinatal outcomes - A randomized controlled trial. *Obstetrics and Gynecology* 2007;110(2):330-9.

27 Ickovics JR, Reed E, Magriples U, et al. Effects of group prenatal care on psychosocial risk in pregnancy: Results from a randomised controlled trial. *Psychology & health* 2011;26(2):235-50.

28 Andersson E, Small R. Fathers' satisfaction with two different models of antenatal care in Sweden - Findings from a quasi-experimental study. *Midwifery* 2017;50:201-7.

29 Ickovics JR, Earnshaw V, Lewis JB, et al. Cluster Randomized Controlled Trial of Group Prenatal Care: Perinatal Outcomes Among Adolescents in New York City Health Centers. *Am J Public Health* 2016;106(2):359-65.

30 Craig P, Dieppe P, Macintyre S, et al. Developing and evaluating complex interventions: the new Medical Research Council guidance. *Int J Nurs Stud* 2013;50(5):587-92.

31 Moore GF, Audrey S, Barker M, et al. Process evaluation of complex interventions: Medical Research Council guidance. *BMJ* 2015;350:h1258.

32 Campbell NC, Murray E, Darbyshire J, et al. Designing and evaluating complex interventions to improve health care. *BMJ* 2007;334(7591):455-9.

33 Yardley L, Morrison L, Bradbury K, et al. The person-based approach to intervention development: application to digital health-related behavior change interventions. *J Med Internet Res* 2015;17(1):e30.

34 Byrskog U, Essen B, Olsson P, et al. 'Moving on' Violence, wellbeing and questions about violence in antenatal care encounters. A qualitative study with Somali-born refugees in Sweden. *Midwifery* 2016;40:10-7.

35 Ahrne M, Schytt E, Andersson E, et al. Antenatal care for Somali-born women in Sweden: Perspectives from mothers, fathers and midwives. *Midwifery* 2019;74:107-115.

36 Lionis C, Papadakaki M, Saridaki A, et al. Engaging migrants and other stakeholders to improve communication in cross-cultural consultation in primary care: a theoretically informed participatory study. *BMJ open* 2016;6(7):e010822.

For peer review only - http://bmjopen.bmj.com/site/about/guidelines.xhtml

37 Johnson CE, Ali SA, Shipp MP. Building community-based participatory research partnerships with a Somali refugee community. *Am J Prev Med* 2009;37(6 Suppl 1):S230-6.

38 Attride-Stirling J. Thematic networks: an analytic tool for qualitative research. *Qualitative Research* 2001;1(3):385-405.

39 Ekman I, Swedberg K, Taft C, et al. Person-centered care - ready for prime time. *Eur J Cardiovasc Nurs* 2011;10(4):248-51.

40 Lundahl B, Moleni T, Burke BL, et al. Motivational interviewing in medical care settings: a systematic review and meta-analysis of randomized controlled trials. *Patient Educ Couns* 2013;93(2):157-68.

41 Wagner CC. Motivational Interviewing in Groups. New York: Guilford Publications; 2013.

42 Sharma J, O'Connor M, Rima Jolivet R. Group antenatal care models in low- and middle-income countries: a systematic evidence synthesis. *Reprod Health.* 2018;15(1):38.

43 Catling CJ, Medley N, Foureur M, Ryan C, Leap N, Teate A, Homer CS. Group versus conventional antenatal care for women. Cochrane Database Syst Rev. 2015;4;(2).

44 Gagnon AJ, DeBruyn R, Essen B, et al. Development of the Migrant Friendly Maternity Care Questionnaire (MFMCQ) for migrants to Western societies: an international Delphi consensus process. *BMC Pregnancy Childbirth* 2014;14:200.

45 Green JM, Kafetsios K, Statham HE, et al. Factor structure, validity and reliability of the Cambridge Worry Scale in a pregnant population. *J Health Psychol* 2003;8(6):753-64.

46 Cox J, Holden J, Henshaw C. Perinatal Mental Health. The Edinburgh Postnatal Depression Scale (EPDS) Manual. 2nd ed: The Royal College of Psychiatrists; 2012.

47 Sudman S, Bradburn NM, Schwarz N. Thinking about answers: the application of cognitive processes to survey methodology. San Francisco: Jossey-Bass Publishers; 1996.

48 Colon-Burgos JF, Colon-Jordan HM, Reyes-Ortiz VE, et al. Disparities and barriers encountered by immigrant Dominican mothers accessing prenatal care services in Puerto Rico. *J Immigr Minor Health* 2014;16(4):646-51.

49 Center for Disease Control and Prevention. Pregnancy Risk Assessment Monitoring System (PRAMS) [Internet]. [Accessed 2016 April 2]. Available from: http://www.cdc.gov/prams/.

50 Liamputtong P. Performing Qualtiative Cross-cultural Research. Cambridge: Cambridge University Press; 2010.

51 Hildingsson I, Rådestad I. Swedish women's satisfaction with medical and emotional aspects of antenatal care. *J Adv Nurs* 2005;52:239-249

For peer review only - http://bmjopen.bmj.com/site/about/guidelines.xhtml

52 Rubertsson C, Wickberg B, Gustavsson P, Radestad I. Depressive symptoms in early pregnancy, two months and one year postpartum-prevalence and psychosocial risk factors in a national Swedish sample. *Arch Women Ment Health* 2005;8(2):97-104.

53 Elo S, and Kyngas, H. The qualitative content analysis process. *J Adv Nurs* 2008 62(1):107-115.

For peer review only

For peer review only

For peer review only - http://bmjopen.bmj.com/site/about/guidelines.xhtml

BMJ Open

Rationale, development and feasibility of group antenatal care for immigrant women in Sweden: a study protocol for the Hooyo-project

Journal:	*BMJ Open*
Manuscript ID	bmjopen-2019-030314.R2
Article Type:	Protocol
Date Submitted by the Author:	16-Jun-2019
Complete List of Authors:	Byrskog, Ulrika; Dalarna University, School of Education, Health and Social sciences<br>Ahrne, Malin ; Karolinska Institute, Department of Women's and Children's Health<br>Small, Rhonda; La Trobe University, Mother and Child Health Research<br>Andersson, Ewa; Karolinska Institute, Department of Women's and Children's Health<br>Essen, Birgitta; Uppsala University, Womens and Childrens Health<br>Adan, Aisha; Karolinska Institute, Department of Women's and Children's Health<br>Ahmed, Fardosa; Karolinska Institute, Department of Women's and Children's Health<br>Tesser, Karin; Antenatal Care Clinic, Domnarvet<br>Lidén, Yvonne; Antenatal Care Clinic<br>Israelsson, Monika; Antenatal Care Clinic<br>Åhman-Berndtsson, Anna; Antenatal Care Clinic, Domnarvet<br>Schytt, Erica; Centre for Clinical Research Dalarna-Uppsala University; Western Norway University of Applied Sciences, Faculty of Health and Social Sciences
**Primary Subject Heading**:	Public health
Secondary Subject Heading:	Public health, Health services research, Obstetrics and gynaecology, Research methods, Patient-centred medicine
Keywords:	Feasibility study, Immigrants, Somali-born women, Process evaluation, Midwives, Group antenatal Care

SCHOLARONE™
Manuscripts

Rationale, development and feasibility of group antenatal care for immigrant women in Sweden: a study protocol for the Hooyo-project

Ulrika Byrskog[a] uby@du.se

Malin Ahrne[b] malin.ahrne@ki.se

Rhonda Small[bc] R.Small@latrobe.edu.au

Ewa Andersson[b] Ewa.Andersson@ki.se

Birgitta Essén[d] birgitta.essen@kbh.uu.se

Aisha Adan[b] Shafeec08@hotmail.com

Fardosa Hassen Ahmed[b] fardosa.hassen.ahmed@ki.se

Karin Tesser[e] karin.tesser@Ltdalarna.se

Yvonne Lidén[f] yvonne.liden@sll.se

Monika Israelsson[f] monika.israelsson@sll.se

Anna Åhman Berndtsson[e] anna.ahman-berndtsson@Ltdalarna.se

Erica Schytt[bgh] Erica.Schytt@Ltdalarna.se

[a] School of Education, Health and Social Studies, Dalarna University, Falun, Sweden

[b] Department of Women's and Children's Health, Karolinska Institutet, Stockholm, Sweden

[c] Judith Lumley Centre, La Trobe University, Melbourne, Australia

[d] Women's and Children's Health, IMCH, Akademiska Hospital, Uppsala Sweden

[e] Antenatal Care Clinic, Domnarvet Borlänge, Sweden

[f] Antenatal Care Clinic, Spånga-Tensta, Sweden

[g] Centre for Clinical Research Dalarna-Uppsala University, Falun, Sweden

[h] Faculty of Health and Social Sciences, Western Norway University of Applied Sciences, Bergen, Norway

Corresponding author: Ulrika Byrskog uby@du.se
Dalarna University, School of Education, Health and Social Studies
791 88 Falun
Sweden

For peer review only - http://bmjopen.bmj.com/site/about/guidelines.xhtml

ABSTRACT

Introduction Somali-born women comprise a large group of immigrant women of childbearing age in Sweden, with increased risks for perinatal morbidity and mortality and poor experiences of care, despite the goal of providing equitable health care for the entire population. Rethinking how care is provided may help to improve outcomes.

Overall aim To develop and test the acceptability, feasibility and immediate impacts of group antenatal care for Somali-born immigrant women, in an effort to improve experiences of antenatal care, knowledge about childbearing and the Swedish health care system, emotional wellbeing and ultimately, pregnancy outcomes. This protocol describes the rationale, planning and development of the study.

Methods and analysis An intervention development and feasibility study. Phase 1 includes needs assessment and development of contextual understanding using focus group discussions. In Phase 2 the intervention and evaluation tools, based on core values for quality care and person-centred care, are developed. Phase 3 includes the historically controlled evaluation in which relevant outcome measures are compared for women receiving individual care (2016-2018) and women receiving group antenatal care (2018-2019): care satisfaction (Migrant Friendly Maternity Care Questionnaire), emotional wellbeing (Edinburgh Postnatal Depression Scale), social support, childbirth fear, knowledge of Swedish maternity care, delivery outcomes. Phase 4, the process evaluation, investigate process, feasibility and mechanisms of impact using field notes, observations, interviews and questionnaires. All phases are conducted in collaboration with a stakeholder reference group.

Ethics and dissemination The study is approved by the Regional Ethical Review Board, Stockholm, Sweden. Participants receive information about the study and their right to decline/ withdraw without consequences. Consent is given prior to enrolment. Findings will be disseminated at antenatal care units, national/international conferences, through publications in peer-reviewed journals, seminars involving stakeholders, practitioners, community and via the project website. Participating women will receive a summary of results in their language.

Keywords: Feasibility study, immigrants, Somali-born women, midwives, group antenatal care, process evaluation

For peer review only

ARTICLE SUMMARY

Strengths and limitations of this study

- The feasibility design of the study together with the nested process evaluation will contribute valuable information for future controlled studies, and in the design of antenatal care interventions that target inequalities in health between immigrants and non-immigrants.
- By emphasising dialogue, a person-centred approach and active participation of parents, midwives and bilingual research assistants throughout development and implementation, recruitment and retention are optimised and a relevant, pragmatic and acceptable model of care is likely to result.
- Funding has limited the scope of the study to two sites, with a focus on one group of immigrant women.

INTRODUCTION

Sweden has a clearly stated goal of providing equitable health care for the whole population, which in recent decades has become increasingly diverse. Despite this, studies indicate that pregnancy outcomes among immigrant women are suboptimal compared with those of Swedish-born women(1). These health disparities point to the need for implementing and evaluating interventions to improve care for immigrant women and their families. This study protocol describes the development of an intervention to improve antenatal care (ANC) for Somali-born women and families giving birth in Sweden.

Somali-born women constitute one of the largest groups of immigrant women of childbearing age in Sweden(2), after more than two decades of political instability in Somalia. Of all immigrant women, they are known to be at high risk of maternal and perinatal morbidity and mortality both in Sweden(3-5) and internationally(6). The reasons are complex and include both pre- and post-migration factors. Insufficient health care provision and low socioeconomic conditions in the country of origin influence women's health status (7). After migration to Sweden, later and lower attendance for antenatal care (8), and poorer experiences of maternity care(9) have been reported. Language difficulties and lack of familiarity with care systems, and discrimination and sub-optimal care are contributing factors(3, 10,11). Furthermore, higher rates of anaemia, insufficient weight gain and infants born small for gestational age(8) indicate that Somali women are not sufficiently

For peer review only - http://bmjopen.bmj.com/site/about/guidelines.xhtml

reached by advice about prevention or treatment. Lower attendance at childbirth and parent education classes among immigrant women has also been found, with classes most often held in Swedish(12).

Few measures have been taken in Sweden to reduce barriers to maternity care for immigrant women or to better meet their care needs. Furthermore, few studies have investigated how care might be designed in ways that are both attractive and acceptable to immigrant women and to midwives who are the main providers of care during pregnancy and childbirth. Respect, communication, community knowledge and care tailored to women's circumstances and needs have been proposed as central components in quality maternity care(13). In line with this, a systematic review in five countries highlighted that immigrant women want the same things from care as non-immigrant women: care should be of high quality and safe, with adequate information and support and attentive to individual needs. The review also found however, that immigrant women consistently reported experiencing additional challenges, including communication difficulties, lack of familiarity with health care systems and discrimination(10).

Language barriers, marginalisation and prevailing cultural stereotypes pose challenges in designing, implementing and evaluating innovative care models including, rather than excluding, immigrants. These factors need to be carefully addressed before widespread change of new models of care are introduced. Participatory approaches have shown promising results(14-16) and reduce the risk of enhancing marginalisation. Focus on communication and meaningful dialogue throughout intervention design, recruitment, data collection, implementation and evaluation is valuable (15).

In Sweden, the care of pregnant women during normal pregnancies is provided at community-based ANC clinics by registered midwives, based on national and regional guidelines(17). Included are 8-10 pregnancy check-ups free of charge, traditionally offered through individual appointments (30 minutes) with the same midwife throughout the pregnancy. Extra visits are booked and specialised medical referrals are made when needed. Parent education including preparation for childbirth and parenting is provided during the individual appointments or in groups/classes, mainly in Swedish and often focusing on first time parents. Partners are encouraged to participate.

One innovative approach for improving antenatal care is group antenatal care (gANC). This care model incorporates pregnancy assessments and parent education in small groups, either with shorter individual appointments with the midwife at the beginning or end of the group session, or with

For peer review only - http://bmjopen.bmj.com/site/about/guidelines.xhtml

individual clinical checks by the midwife provided in the group space (18-20), while the rest of the group can socialise. Self-assessments of blood pressure and urine have also been included in some gANC models(18, 20). Results from studies in the US, Iran and Sweden report improved satisfaction with care(19, 21-24), improved iron taking, higher birthweight, lower caesarean section rates, more breastfeeding and earlier diagnosis of complications(23). Higher self-esteem and lower levels of stress, social conflicts and depression have been reported, as have more visits to ANC by fathers-to-be, increased support in social contacts with other parents, and reduced costs(18, 19, 23, 25-28), despite more time spent with the midwife throughout pregnancy. gANC thus appears to enhance women's agency, dialogue, time with the midwife and social interaction, and to improve women's views of their care and also some of their pregnancy outcomes. gANC may be especially valuable for specific groups of women, as seen for example in studies among young African-American women in the United States(26, 29) and among Karen refugee women in Australia(15).

The *Hooyo-project* ('mother' in Somali) is a gANC initiative developed in Sweden in response to the evidence about lack of equity in pregnancy care provision and poorer outcomes for Somali-born women. gANC has not been evaluated among immigrant women and families in Sweden, yet it may well be a model of care that can break down cultural and care barriers, enable better dialogue and understanding between caregivers and immigrant women and improve women's satisfaction with their care, especially if language support is provided(15).

The overall aim for this study is to develop and test the acceptability, and immediate impacts of gANC for Somali women residing in Sweden, in an effort to improve experiences of antenatal care, knowledge about childbearing and the Swedish health care system, emotional wellbeing and ultimately, pregnancy outcomes.

Specific objectives include

1) To establish with Somali-born women and antenatal caregivers the acceptability and feasibility of gANC and to develop an appropriate model in order to improve outcomes (Phases 1 and 2);

2) To establish appropriate recruitment and data collection procedures and outcome measures in consultation with the Somali community and with care providers in order to evaluate the new model (Phase 2) ; and

3) To implement and evaluate an agreed model of gANC in partnership with the Somali community and antenatal caregivers (Phases 3 and 4).

For peer review only - http://bmjopen.bmj.com/site/about/guidelines.xhtml

METHODS AND ANALYSIS

Design

An intervention development and feasibility study including focus group discussions, a historically controlled evaluation and process evaluation(30, 31). The intervention is implemented in two antenatal care clinics in mid-Sweden. The feasibility and piloting includes testing procedures for acceptability, estimating the likely rates of recruitment and retention of women, and the calculation of appropriate sample sizes for future controlled trials. Complex interventions that are adjusted to local, specific(32) or personal needs(33) are likely to be more effective than completely standardised models(30). Key principles underpinning the study are therefore:

- Active involvement of Somali parents and midwives in needs assessment and care design
- Attention to language and contextual factors
- Flexibility in study methods to respond to issues as they arise
- A care model ready to continue or be replicated after the project concludes with only minor adjustments.

Hooyo consists of four phases with process evaluation activities informing study progress. *Phase 1* is the preparation phase, including needs assessment, the development of contextual understanding and building a logic model for the project. *Phase 2* includes the development of the intervention and of the evaluation tools and *Phase 3* involves implementation and evaluation of the intervention using historical controls. In *Phase 4* the implementation process, the feasibility and the mechanisms of impact are evaluated, including contextual factors, using Moore's Process Evaluation of Complex Interventions as an overarching framework(31). Phases 1 and 2 were conducted in 2016-2017. Recruitment and data collection for the historically controlled evaluation (Phase 3) and process evaluation (phase 4) commenced in late 2016 and will be completed during 2020. Women in the control group were recruited between October 2016 and April 2018 and recruitment of women into the intervention began in May 2018 and will be complete in December 2019. Final data collection will be completed by July 2020. The paper covers methodological aspects of all four phases of the study. The paper covers methodological aspects of all four phases of the study.

For peer review only - http://bmjopen.bmj.com/site/about/guidelines.xhtml

Phase 1 – Preparation phase

The purpose of Phase 1 was to understand how current individual care was delivered and experienced and what changes women, partners and midwives thought might improve care, taking into account contextual factors that might have an impact on the development, structure and content of a new model. The aim was also to assess whether a language-supported group antenatal care model might be appropriate and acceptable to address concerns raised about current care.

Initial dialogue and choosing study settings

In the municipality chosen for *site one,* the number of Somali immigrants had increased fivefold during the last decade and the ANC midwives had begun to re-think how they were providing care. At the same time, there was awareness of lower ANC attendance and adverse outcomes among child-bearing Somali-born women in Sweden(8), and a need for more appropriate explanations of ANC routines had been highlighted in interviews with Somali-born women(34). This prompted a dialogue between the principal investigator and the ANC midwives, outlining the possibility of evaluating an alternative model of care. The ANC clinic is located in a Primary Health Care Centre staffed by 10 midwives, within a public hospital located outside the city centre. It caters for approximately 75% of all pregnant women in the municipality and almost all of the pregnant immigrant women in the municipality, which in total has 50,000 residents. The majority of the Somali-born women visiting the clinic live in two areas situated approximately five kilometres away, necessitating use of a car or public transport.

At *site two*, the ANC clinic is located in a Family Health Centre in a suburb of the capital city. Close collaboration takes place between ANC midwives, social workers, child health nurses and the open playgroup located in the same building. Three ANC midwives are employed and the target area includes two residential areas with different socio-demographics; with primarily Swedish-born residents in one area and non-Swedish born residents and second generation immigrants in the other. Somali-born residents have lived in this area for more than 25 years and midwives reported that most Somali-born pregnant women and families were able to communicate in Swedish. Integration of families of different cultural backgrounds is actively encouraged at the clinic. Initial discussions revealed that the midwives believed group antenatal care for women of mixed cultural backgrounds (with interpreters available if needed) would contribute positively to integration. As a result, it was agreed that integrated groups would be more appropriate and acceptable at this site.

For peer review only - http://bmjopen.bmj.com/site/about/guidelines.xhtml

For peer review only

Mechanisms for engagement: Reference group and bicultural research assistants

A project reference group was established comprising research team members, antenatal care midwives and representatives of the Somali community from both sites. Terms of reference were developed, including provision of advice to the research team on design of the intervention, appropriate recruitment processes and data collection methods; development of study questionnaires; networking with Somali associations and ANC clinics; as well as contributing to the interpretation and dissemination of findings throughout the project period.

A bilingual Somali research assistant with a health care background was employed full time in the project at site one. This enabled networking with Somali community members, bridging language gaps, input into questionnaire design, recruitment of participants, data collection and arranging and interpreting focus group discussions together with research team members. At site two, focus group discussions were facilitated by two Somali community workers, including recruitment and interpreting.

Participatory focus group discussions with Somali parents and antenatal care midwives

To develop understanding of current antenatal care and how it was experienced, Phase 1 included focus group discussions (FGDs) with Somali-born parents and with ANC midwives; presented in brief below and in Ahrne et al(35).

Parents were recruited using purposeful sampling. In total, 16 mothers and 13 fathers with varied length of stay in Sweden and recent experience (<2 years) of ANC were included. Seven ANC midwives were recruited purposefully from both sites and from an additional site where the midwives had previous experience of group antenatal care with Somali-born parents.

Three focus group discussions were held with Somali-born mothers, two with Somali-born fathers and three with ANC midwives. Emerging themes were highlighted by the researchers and crosschecked during the discussions with participants for accuracy(36, 37). Thematic analysis as described by Attride-Sterling(38) was used to interpret data from the FGDs.

Key findings informing intervention development

Challenges at the system level and in the care encounter in striving for optimal ANC were identified(35); alongside findings that could directly inform the intervention development. Instead of focusing on specific cultural or ethnic aspects of care, the results indicated a need to maintain focus on diversity and individual needs. The need for forums for social interaction, and for dialogue

For peer review only - http://bmjopen.bmj.com/site/about/guidelines.xhtml

which could bridge gaps between midwives' and women's understanding of the purpose of antenatal care, improve communication and reduce the risks for stereotyping was apparent. Responding to a wide range of health literacy needs, welcoming fathers and safeguarding an open atmosphere were other issues raised. Furthermore, practical issues such as optimal location and times for ANC visits were discussed. The findings contributed valuable input to the program theory and to the decisions regarding underpinning central principles for the coming intervention (Box 1), as well as for the development of the structure and content of the intervention in Phase II.

For peer review only

For peer review only - http://bmjopen.bmj.com/site/about/guidelines.xhtml

Problem statement	Conceptual framework and rationale	Hooyo: A group approach to improving ANC	Hypothesised mechanisms of effect	Desired outcomes
**Current ANC in Sweden may not provide equitable care for Somali-born women:**<br>• Lower participation in antenatal care<br>• Poorer birth outcomes<br>• Communication difficulties<br>• Lack of familiarity with Swedish antenatal care structures<br>• Lower attendance in parental education<br>• Negative attitudes and suboptimal care<br>**Initial FGDs with Somali-born parents/ANC midwives highlight need for:**<br>• Improved communication and dialogue<br>• Bridging gaps between divergent health literacy knowledge<br>• Care free from generalisa-tions, tailored to individual needs<br>• Clearly described expecta-tions regarding partner's role	**Core values for quality care:** respect, communication, community knowledge and understanding<br><br>**Person-centred care** to identify and address women's individual needs<br>**Continuity of care** for positive care experiences and health outcomes<br><br>**Group antenatal care** a promising alternative to individual visits:<br>• More positive views of care<br>• Some positive impacts on birth outcomes<br>• More time with midwives and more comprehensive parental education<br>• In Sweden studied with Swedish-speaking groups only<br><br>**Key underpinning principles**:<br>• Active involvement of Somali parents/ midwives in assessment and care design<br>• Attention to language and contextual factors<br>• Flexibility in study methods to respond to issues as they arise<br>• A care model ready to continue or replicate after project ending with minor adjustments	**Language supported group antenatal care,** involving<br>• 8-9 group sessions 1 1/2 hours with 6-8 women (partners welcome) from gest. week 24<br>• Facilitated by two midwives assisted by interpreter<br>• Brief individual midwife check-ups incorporated<br>• Childbirth/parenting themes with focus on dialogue and discussion<br>• Motivational interviewing for groups as a vehicle for focusing care on women's needs<br>• Adjustments based on local needs at each site:<br>*Site 1:* Groups specifically for Somali-born<br>*Site 2*: Groups with diverse backgrounds and languages	**Interpreter-supported group dialogue facilitated by midwives will result in**<br>• Improved communication →better suited care<br>• More time for discussions →mutual understandings in views around childbirth and health promotion → strategies for improving outcomes<br>• An additional arena for social contact and support →increased well-being<br>• Combining pregnancy check-ups with groups →motivation for attending ANC, and parental education<br>• Common language/ background → understanding and empower women to raise voices in having needs addressed<br>• Mixed groups →integration and understanding through cross-language/culture interactions	**Women:**<br>• Happier with the ANC<br>• More confident in and knowledgeable about the pregnancies<br>• Improved wellbeing<br>• Improved attendance at antenatal care visits<br>• Improved uptake of health advice<br>• Ultimately; improved pregnancy outcomes<br><br>**Partners:**<br>• Feeling welcomed and included<br>• Increased understanding of expectations<br><br>**Midwives**:<br>• Improved understanding of womens needs<br>• Feel better able to share health knowledge in meaningful ways<br>• Provide more supportive, non-judgemental care<br>• Positive about benefits of group care

Box 1. Logic model of Hooyo including problem statements, conceptual framework and rationale, the Hooyo group antenatal care model, hypothesised mechanisms of effect and desired outcomes.

For peer review only - http://bmjopen.bmj.com/site/about/guidelines.xhtml

Phase 2 – Development of the intervention and study evaluation tools

Developing group antenatal care

Prior to the study commencing, a review of the literature on migrant women's birth outcomes, experiences of antenatal care and on alternative models of care for migrant women had been undertaken, including group antenatal care. This, together with the experiences of team member EA in evaluating group antenatal care in Sweden(19, 24, 28), led us to investigate whether group antenatal care could be appropriately developed to improve outcomes for Somali-born women. The preliminary findings from the FGDs confirmed language supported gANC as a possible alternative care model and this was discussed in the Reference Group. Research team members participated in discussions at each study site during these processes. The intervention development was characterised by constant modifications to fit local prerequisites at both sites, including local guidelines and routines in the ANC clinics, and needs among the women; not all of which were apparent from the start.

Underpinning principles

Central findings in the FGDs were the desire for individualised care, a need for forums for dialogue to address variations in health literacy and health care understanding, and at the same time respond to the request to move beyond stereotypes based on ethnicity or "culture". This led us to focus on Person Centred Care (PCC)(39) as a foundation for the intervention, and on how this could be strengthened and encouraged through active dialogue in group-based care. This was also in line with the ongoing implementation of PCC generally in the health system in Sweden. In searching for a method to support this approach, the midwives proposed Motivational Interviewing (MI). They had already received some training in MI for use in individual appointments, and now MI *for groups* was added. Principles of person-centred care and MI encourage understanding the person as an individual, and developing partnership and promoting self-efficacy for which an active and open dialogue is central(39-41).

Addressing language and integration

To respond to the diverse needs identified at the two ANC clinics and to assess advantages and disadvantages with both homogeneous and heterogeneous groups regarding language and cultural background, it was decided that gANC would be offered to Somali-born women at site 1. A large number of Somali migrants had settled in this municipality in recent years and this meant that many were not yet fluent in Swedish. At site 2, gANC would be offered to *all* women attending the clinic,

For peer review only - http://bmjopen.bmj.com/site/about/guidelines.xhtml

in integrated groups. At site 1, the need for a Somali interpreter at each session was anticipated. Since a finding in the FGDs was that continuity of known Somali interpreters had been a previous success factor, it was decided to engage two experienced interpreters who could alternate. At site 2 it was anticipated that the need for interpreters in different languages would vary and thus they would be engaged when needed.

The intervention: Group Antenatal Care (gANC)

The intervention consists of gANC offered at both sites, modified to the needs in each site and group. Women are allocated to a group with women at similar stages of pregnancy within a four-week gestational age span. From the second visit (gestational week 20-26) they receive group antenatal care. Each group will consist of 6-8 women and partners (or another support person) for optimal group dynamics and dialogue. While reports of gANC internationally involve numbers in groups of 5-20 in low to middle income countries (42) and 8-12 in high income countries (43), the need to provide interpreting during groups sessions led to the choice of a somewhat smaller group size as being appropriate to allow all group members a chance to participate in the discussions. Partners are generally encouraged to participate in Swedish ANC, and hence this will be the point of departure also for the gANC, but with the final decision about partner participation taken by the women included in each group. Frequency and number of sessions follow the national Swedish program for antenatal care, i.e. 8-9 appointments during a normal pregnancy. Each visit includes a group session for one hour, facilitated by one of two midwives assigned to the group, and with interpreter assistance. Although each group session has a focus on a theme related to pregnancy, birth or parenting in line with national recommendations(17), particular emphasis will be given to issues and questions raised by the participants. Group care allows women to talk with each other and share their experiences in their own language. They also hear midwives' responses to other women's questions, expanding the dialogue and information sharing that can occur between midwives and women. The presence of an interpreter facilitates communication between the women and the midwives.

Alongside the group discussion each woman also has a 15-minute individual appointment with the midwife responsible for her care, who is the same midwife for each appointment according to Swedish National Guidelines(17). Routine pregnancy controls are carried out and the woman can also raise any particular issues with her midwife during this time. In total the time for each visit will be approximately 75 minutes instead of 30 minutes, as is common for standard individual care (though this does not take into account the time midwives also spend providing childbirth and

For peer review only - http://bmjopen.bmj.com/site/about/guidelines.xhtml

parenting classes). Additional individual appointments can be booked as needed if medical or other issues arise.This model for group antenatal care draws on the model used in a previous Swedish study by Andersson et al(19, 24, 28), but differs in that two midwives and a language interpreter are present throughout the group sessions and the principles of PCC and MI are explicitly identified as the theoretical underpinnings for care in the group.

Development of Evaluation Tools

Questionnaires

Questionnaires for data collection from participating women were developed by the research team. A number of questions were taken from the Migrant Friendly Maternity Care Questionnaire (MFMCQ)(44), in some cases slightly modified to fit the purpose of our study, as is the intention in use of the MFMCQ, and for use both in pregnancy and after birth, including occasional changes of tense from the original. Selected questions were also included from the Cambridge Worry Scale (11 single-item questions on worries about the upcoming labour and birth), which is available in English and Swedish(45), and the ten-item Edinburgh Postnatal Depression Scale (EPDS) which is available in English, Swedish and Somali(46). The questionnaires were developed in English by the research team and translated to Swedish by one of the bilingual team members and then cross-checked by another bilingual researcher in the team. Thereafter the questionnaire was translated into Somali by a professional Somali translator, fluent in English, Swedish and Somali, and finally cross-checked by a Somali-speaking research assistant. At each step of the process the questionnaire was pilot-tested with Somali-born women for relevance and understanding with adjustments made accordingly. During piloting it was clear that some of the items and response alternatives in the EPDS were difficult for women. Although the scale is translated into Somali, no validation study has to date been published. As data are collected in face-to-face or telephone interviews conducted by the bilingual research assistant, a decision was made to include the EPDS as translated, with the research assistant able to explain the meaning of the questions in plain language if needed. Alongside the current study, we have commenced a process for validating the EPDS Somali translation, involving think-aloud interviews(47) with Somali-born women, in order to contribute to evidence about use of the EPDS in Somali.

Outcome measures

Primary outcomes are women's overall ratings of antenatal care and emotional wellbeing. Ratings of care are assessed by the core question "When thinking about your overall experience of antenatal

For peer review only - http://bmjopen.bmj.com/site/about/guidelines.xhtml

care - in general, have you been happy with the care that you have received?" with response alternatives *always, mostly, sometimes, rarely and never*. The question is modified from the Migrant Friendly Maternity Care Questionnaire (MFMCQ)(44) and more detailed questions about specific aspects of care are also included. Emotional wellbeing is assessed with the Edinburgh Postnatal Depression Scale (EPDS), completed in English, Swedish or Somali as appropriate (46).

Secondary outcomes are adequate number of visits measured by the Adequacy of Prenatal Care Utilization Index (APCUI), i.e. expected number of visits in relation to actual number adjusted for gestational week at booking visit and delivery(48), social support during pregnancy using slightly modified questions from the Pregnancy Risk Assessment Monitoring System (PRAMS)(49) and worries about the upcoming birth using questions from the Cambridge Worry Scale(45). Knowledge about danger signs and where to seek health care if particular symptoms arise are assessed by questions with multiple choice responses, such as "Now at the end of your pregnancy, where would you turn if you had bleeding from the vagina? When would you seek care?". From the patient record, the following are retrieved: estimated time spent with the antenatal care midwife, gestational age at the first ANC visit, lowest measurements of Haemoglobin and S-Ferritin, weight gain, attendance at parent education, mode of birth, interventions (induction of labour, pain relief including epidural, oxytocin), blood loss, diagnoses (mother) and gestational age, birth weight, Apgar score, umbilical cord pH, and the need for neonatal intensive care (infant).

Preparing for implementation

Awareness raising

To create awareness and interest in the project and subsequently in the new model of care, Somali and other community networks are involved in different ways. Information is disseminated by participants in the reference group, by researchers and research assistants one-to-one, and in small groups gathering Somali-born women. A brief pamphlet in Swedish and Somali has been produced and distributed among women, Somali community associations, open playgroups and activity centres and as a poster in the waiting rooms at participating ANC clinics.

Training of midwives and interpreters

Areas to be covered in a preparatory training workshop for midwives were identified through findings in the FGDs, in dialogue with the midwives at both sites and in reference group meetings. The research team then tailored a 1 ½ day workshop for all midwives at both sites, held prior to intervention commencement. In response to the needs expressed, the main focus was group

For peer review only - http://bmjopen.bmj.com/site/about/guidelines.xhtml

processes including person-centred care(38) and motivational interviewing (MI) in groups(40). A two-day follow-up session is planned during the intervention period to enable feedback on implementation and address any issues arising.

The interpreters' preparation included two hours of information regarding the structure of the ANC care system, introduction to the Hooyo-project and the gANC model emphasising dialogue and MI-principles, participation in meetings with involved midwives and receiving a written project manual (described below).

Development of material for midwives leading group sessions

A manual comprising information and suggestions for the content and structure of group sessions has been developed, which the midwives are free to use and adjust according to the needs in the groups. An overview of the availability of evidence-based resources related to pregnancy and birth led us to recommend the use of existing resources where available, as they are free of charge and often in different languages. This also adds to the sustainability and cost-effectiveness of the intervention, and makes it easier to duplicate. A study-specific homepage was created, comprising the same information and support as in the written manual.

Phase 3 – Design and methods for the historically controlled evaluation of the intervention

Participants and recruitment

At both sites, primiparous and multiparous Somali-born women are recruited to the study. Recruitment and retention of participants to clinical studies is a well-documented challenge in research across languages and cultures(50). We therefore thoroughly planned how to optimise the recruitment and information processes in relation to eligible women and adjusted processes to local prerequisites at both sites. The Somali-speaking research assistant is based at site 1 where most needed, and where the majority of the recruitment is performed. In site 2, team-member MA is involved with recruitment, information and data collection, supported by the Somali-speaking research assistant form site 1. All pregnant Somali-born women attending the two ANC clinics receive brief information about the study from the midwives during their first visit and if they agree to receive further information, they are then contacted by the research assistant or MA after the first ANC visit, either by telephone or face to face for further oral and written information in Somali, English or Swedish, as needed.

For peer review only - http://bmjopen.bmj.com/site/about/guidelines.xhtml

Inclusion criteria are being born in Somalia and <25 weeks of gestation. *Exclusion criterion* is a health condition preventing participation in group antenatal care, i.e. severe mental health condition or if the pregnancy care were to be transferred to a specialist obstetric clinic. During the first 18 months of the study, recruited women were included in the control group receiving standard individual care and in the following 18 months women are included in the intervention group. All women are informed verbally and orally about the purpose of the study and that participation is voluntary. Women included in the control group were informed that their perspectives about their care would contribute knowledge valuable for a proposed intervention study aimed at improving antenatal care for migrant women.

Data collection for the intervention

Questionnaires

Questionnaire data are collected at three time points (Q1, Q2, Q3). The baseline measurement (Q1) is conducted after recruitment, i.e. before gestational week 25. Women are contacted for a face-to-face or telephone structured interview in Somali, Swedish or English at a place of their choice. Q1 includes questions on background (parity, education, income, occupation), migration (time of residence, reason for migration, migration status, language) and social support, psychological and physical wellbeing, knowledge of danger signs and the health system, and expectations about the up-coming birth. Data for Q2 are collected in late pregnancy, after gestational week 35, in the same manner. Q2 includes questions on experiences of care and information during the ongoing pregnancy, knowledge of the health system, pregnancy and birth, social support, and psychological and physical wellbeing and expectations for the upcoming birth. Q3 is administered two months postpartum and includes questions regarding the overall experience of pregnancy, ANC, birth and psychological and physical wellbeing. After each interview, a small gift is offered as a token of appreciation for women's participation.

Medical records

Data for secondary outcome measurement will be collected from patient medical records, for all consenting participants. This includes data related to pregnancy, labour and birth and maternal and infant outcomes.

For peer review only - http://bmjopen.bmj.com/site/about/guidelines.xhtml

Sample size issues

Our main purpose is to develop and test the feasibility of the intervention and study processes and in so doing, also provide information to inform sample size calculations for possible future randomised controlled trials. Nevertheless, based on available birth rate data we estimate being able to include 100 women in the control group and 100 women in the intervention group during the study period. We estimate that the study will have the power to detect a clinically relevant increase in women's overall satisfaction with antenatal care from an expected 65% among Somali women receiving individual care to 82% in those receiving gANC (approximating rates for Swedish speaking women in a national population based study(51) with 80% power and an alpha of 20%, with 70 women in each group. To have similar power to detect differences in mean scores on the Edinburgh Postnatal Depression Scale (a hypothesised reduction from a mean of 8.0 in the control group – similar to that found in studies of migrant women, to 6.0 in the gANC group – similar to that found in Swedish population-based studies(52), 63 women are required in each group. Allowing for 20% loss to follow-up at the time of data collection with women two months postpartum, 174 women will need to be recruited.

Differences between groups will be described as odds ratios and 95% confidence intervals after estimation by means of logistic regression analyses. Comparison of means will be undertaken using t-tests where data are normally distributed, or medians compared using Mann-Whitney U tests if not.

Phase 4 –Design of the process evaluation

A process evaluation using a mixed methods approach will be conducted throughout the study (30). The process evaluation will address whether the intervention was carried out according to plan, what was achieved, if and what adaptations were needed, who was reached by the intervention, any unexpected events and will also aim to understand the mechanisms of impact and what mediated these mechanisms. The process evaluation will also address contextual factors(31).

Process evaluation data collection and measures

Data collection for the process evaluation(31) will be nested in the different phases, supported by different measurement tools and activities. Moore's process evaluation framework(31) will guide the data collection, analysis and interpretation. The framework emphasises the impact of, and relationship between the intervention, implementation, mechanisms and context, and a topic guide and analysis will focus on *what* is achieved and *how* (fidelity, dose, adaptations, reach). Focus for

For peer review only - http://bmjopen.bmj.com/site/about/guidelines.xhtml

the mechanisms of impact will be the participants' interactions with the intervention and potential mediating factors.

A session checklist for completion by the midwife after each group session will cover topics discussed, resources used, number of participating women and support people, group dialogue and the midwife's brief view of each session. The checklist was developed by the research team, discussed with the reference group and, after adjustments, presented to all included midwives for further modifications, which were addressed by the researchers before intervention start.

Participant observations during the group sessions will contribute data describing the delivery of gANC and the mechanisms of impact by describing group dynamics, dialogue and active participation by women in group sessions. A protocol for observations during group sessions developed by the research team, focuses on group mechanisms, dialogue and interaction, based on PPC and MI principles. This will provide the guide for participant observations by one or two members of the research team during a number of randomly selected group sessions.

Participant experiences of group antenatal care. Questionnaire data for all participating women and qualitative data collected from women, midwives, partners, interpreters and heads of departments will describe responses to and interactions with the intervention and their views regarding the feasibility of the intervention and mechanisms of impact. Women's experiences of the content, structure and feasibility of gANC will be assessed in the *follow-up questionnaire* (Q3) at 2 months postpartum. Additionally, the questionnaires will identify a sample of women with a diversity of experiences to be recruited for *more in-depth individual interviews*. Midwives' experiences of the same, and their perceptions regarding mechanisms of impact will be collected through *qualitative FGDs or interviews* as appropriate. Partners' and interpreters' views will be collected through *qualitative individual interviews* upon completion of participation. FGD and interview data will be collected using a topic guide with open-ended questions developed by the research team and analysed using deductive content analysis according to Elo and Kyngas(53). *Field notes* taken by the researchers in dialogue with research assistants, involved midwives and heads of departments will assist in describing the choices and decisions made during the implementation process.

Patient and public involvement

The initial literature review informing the research questions included studies focusing on immigrant women's experiences and views of maternity care post-migration, including the views of Somali-born women. Initial focus group discussions for the study included Somali women and men

For peer review only - http://bmjopen.bmj.com/site/about/guidelines.xhtml

with recent experiences of antenatal care and these informed the development of the intervention. As described above, a study reference group involves Somali community members in all stages of the project and a bicultural research assistant is involved in recruiting women to the study. All participants will receive a summary of the results in their preferred language and seminars will be held with stakeholder groups.

DISCUSSION

The Hooyo study is an attempt to address some of the health and care disparities in the Swedish antenatal care system. The development and feasibility design of the study(30) together with the nested process evaluation(31) will contribute valuable information for future randomised controlled studies, as well as in the design of antenatal care interventions focused on reducing inequalities in health between immigrants and non-immigrants. The study will provide guidance on the acceptability of this model of care among Somali-born women, their partners and midwives in Sweden, and on recruitment and data collection from responders who are often excluded from research due to communication barriers or marginalisation. The study will also indicate whether gANC has a positive impact on Somali women's experiences of antenatal care and might therefore be appropriate for other migrant groups.

For the midwives, the intervention provides a platform for reflection and dialogue about improving care for migrant women. This is vital, since care provision is always an interplay between care-givers and care-receivers. The early inclusion of midwives' and participants' views provided space to shape the planned intervention in relevant ways in terms of content, structure and underlying core principles(33). By emphasising dialogue, a person-centred approach and the active participation of women, partners and midwives throughout development and implementation of the intervention, we hope to develop a relevant, pragmatic and acceptable model of care for this group of immigrant women, and one that might replicate well to other settings and groups with minor adjustments. The challenge is to develop and implement an acceptable model of care addressing diverse needs of the care-receiving group and suited to local conditions, while ensuring that medical and public health guidelines are fulfilled. The findings of the study will determine if this can be achieved.

It could be argued that the study should include more antenatal care sites and a range of different immigrant groups. Funding limitations meant restricting the study to two sites and focusing on only one immigrant group, something that may limit the study's generalisability. On the other hand, it is

For peer review only - http://bmjopen.bmj.com/site/about/guidelines.xhtml

expected that many of the strategies employed to develop and test group antenatal care in this study, and the lessons learned in doing so, will have future relevance in improving antenatal care for immigrant women more generally.

ETHICS AND DISSEMINATION

The study is approved by the Regional Ethical Review Board, Stockholm, Sweden, 2015-12-04, Dnr 2015/1703-31/1. All participants are offered information about the study, informed about the voluntary nature of participation in detail and give their written consent prior to enrolment.

The findings of the study will be disseminated at relevant national and international conferences and through publications in peer-reviewed journals. Seminars involving local stakeholders in the communities, county council representatives and practitioners will further provide a platform for dissemination and reflections about lessons learned at the end of the project. The findings of the study will also be presented via the Hooyo-project website https://ki.se/kbh/modrahalsovard-for-utlandsfodda-kvinnor-hooyo-projektet.

DECLARATIONS

Acknowledgements
The authors would like to thank the midwives, members of the Hooyo reference group and all the Somali-born women and men who have contributed in the preparation phase of the study.

Consent for publication

Not applicable.

Availability of data and material

The dataset generated and analysed during the preparation phase of the current study is not publicly available due to restrictions based on Swedish law safeguarding individual privacy. It may be made available upon reasonable request.

Competing interests

The authors declare that they have no competing interests.

Funding

This study has received funding from the Swedish Research Council [grant number 2015-02470], Forte [grant number 2016-00957] and the Doctoral School in Health Care Sciences, Karolinska

For peer review only - http://bmjopen.bmj.com/site/about/guidelines.xhtml

Institutet [grant number 2-144/2016]. The funding bodies have played no role in the design of the study, nor in data collection, analysis and interpretation of the data or in writing the manuscript.

Author statement

ES, RS, EA and BE initiated the study. UB, ES, RS, EA MA, BE, AA, FH, KT, YL, MI and AB contributed to planning and design. UB, MA, ES, RS, EA AA and FH developed questionnaires and topic guides. UB drafted the manuscript, MA, ES, RS, UB, EA, BE, AA, FH, KT, YL, MI and AB revised it. All authors read and approved the final manuscript.

REFERENCES

1 National Board of Health and Welfare. Socioekonomiska faktorers påverkan på kvinnors och barns hälsa efter förlossning. Stockholm: National Board of Health and Welfare; 2016.

2 Statistics Sweden [Internet]. [Accessed 2018 Aug 20]. Available from: http://www.statistikdatabasen.scb.se/.

3 Esscher A, Binder-Finnema P, Bodker B, et al. Suboptimal care and maternal mortality among foreign-born women in Sweden: maternal death audit with application of the 'migration three delays' model. *BMC Pregnancy Childbirth* 2014;14:141.

4 Essen B, Bodker B, Sjoberg NO, et al. Are some perinatal deaths in immigrant groups linked to suboptimal perinatal care services? *BJOG* 2002;109(6):677-82.

5 Essen B, Hanson BS, Ostergren PO, et al. Increased perinatal mortality among sub-Saharan immigrants in a city-population in Sweden. *Acta Obstet Gynecol Scand* 2000;79(9):737-43.

6 Small R, Gagnon A, Gissler M, et al. Somali women and their pregnancy outcomes postmigration: data from six receiving countries. *BJOG* 2008;115(13):1630-40.

7. Ali AM, Handuleh J, Patel P, et al. The most fragile state: healthcare in Somalia. *Med Confl Surviv*. 2014;30(1):28-36.

8 Rassjo EB, Byrskog U, Samir R, et al. Somali women's use of maternity health services and the outcome of their pregnancies: a descriptive study comparing Somali immigrants with native-born Swedish women. *Sex Reprod Healthc* 2013;4(3):99-106.

9 Berggren V, Bergstrom S, Edberg AK. Being different and vulnerable: experiences of immigrant African women who have been circumcised and sought maternity care in Sweden. *J Transcult Nurs* 2006;17(1):50-7.

For peer review only - http://bmjopen.bmj.com/site/about/guidelines.xhtml

10 Small R, Roth C, Raval M, et al. Immigrant and non-immigrant women's experiences of maternity care: a systematic and comparative review of studies in five countries. *BMC Pregnancy Childbirth* 2014;14:152.

11 Binder P, Borne Y, Johnsdotter S, et al. Shared Language Is Essential: Communication in a Multiethnic Obstetric Care Setting. *J Healthc Commun* 2012;17(10):1171-86.

12 Fabian H, Radestad I, Rodriguez A, et al. Women with non-Swedish speaking background and their children: a longitudinal study of uptake of care and maternal and child health. *Acta paediatrica* 2008;97(12):1721-8.

13 Renfrew MJ, McFadden A, Bastos MH, et al. Midwifery and quality care: findings from a new evidence-informed framework for maternal and newborn care. *Lancet* 2014;384(9948):1129-45.

14 Osman F, Salari R, Klingberg-Allvin M, Schon UK, et al. Effects of a culturally tailored parenting support programme in Somali-born parents' mental health and sense of competence in parenting: a randomised controlled trial. *BMJ open* 2017;7(12):e017600.

15 Riggs E, Muyeen S, Brown S, , et al. Cultural safety and belonging for refugee background women attending group pregnancy care: An Australian qualitative study. *Birth* 2017;44(2):145-52.

16 Riggs E, Yelland J, Szwarc J, et al. Promoting the inclusion of Afghan women and men in research: reflections from research and community partners involved in implementing a 'proof of concept' project. *Int J Equity Health* 2015;14:13.

17 The Swedish Society of Obstetrics and Gynecology (SFOG) and The Swedish Association of Midwives. Mödrahälsovård, Sexuell och Reproduktiv Hälsa. 2016; ARG report 76.

18 Lathrop B. A systematic review comparing group prenatal care to traditional prenatal care. *Nurs Womens Health* 2013;17(2):118-30.

19 Andersson E, Christensson K, Hildingsson I. Mothers' satisfaction with group antenatal care versus individual antenatal care - a clinical trial. *Sex Reprod Healthc* 2013;4(3):113-20.

20 Hunter L, Da Motta G, McCourt C, et al. `It makes sense and it works': Maternity care providers' perspectives on the feasibility of a group antenatal care model (Pregnancy Circles). *Midwifery* 2018;66:56-63.

21 Ickovics JR, Kershaw TS, Westdahl C, et al. Group prenatal care and perinatal outcomes: a randomized controlled trial. *Obstet Gynecol* 2007;110(2 Pt 1):330-9.

22 Jafari F, Eftekhar H, Mohammad K, et al. Does Group Prenatal Care Affect Satisfaction And Prenatal Care Utilization in Iranian Pregnant Women? *Iran J Public Health* 2010;39(2):52-62.

For peer review only - http://bmjopen.bmj.com/site/about/guidelines.xhtml

23 Jafari F, Eftekhar H, Fotouhi A, Mohammad K, Hantoushzadeh S. Comparison of maternal and neonatal outcomes of group versus individual prenatal care: a new experience in Iran. *Health Care Women Int* 2010;31(7):571-84.

24 Andersson E, Christensson K, Hildingsson I. Parents' experiences and perceptions of group-based antenatal care in four clinics in Sweden. *Midwifery* 2012;28(4):502-8.

25 Wedin K, Molin J, Crang Svalenius EL. Group antenatal care: new pedagogic method for antenatal care--a pilot study. *Midwifery* 2010;26(4):389-93.

26 Ickovics JR, Kershaw TS, Westdahl C, et al. Group prenatal care and perinatal outcomes - A randomized controlled trial. *Obstetrics and Gynecology* 2007;110(2):330-9.

27 Ickovics JR, Reed E, Magriples U, et al. Effects of group prenatal care on psychosocial risk in pregnancy: Results from a randomised controlled trial. *Psychology & health* 2011;26(2):235-50.

28 Andersson E, Small R. Fathers' satisfaction with two different models of antenatal care in Sweden - Findings from a quasi-experimental study. *Midwifery* 2017;50:201-7.

29 Ickovics JR, Earnshaw V, Lewis JB, et al. Cluster Randomized Controlled Trial of Group Prenatal Care: Perinatal Outcomes Among Adolescents in New York City Health Centers. *Am J Public Health* 2016;106(2):359-65.

30 Craig P, Dieppe P, Macintyre S, et al. Developing and evaluating complex interventions: the new Medical Research Council guidance. *Int J Nurs Stud* 2013;50(5):587-92.

31 Moore GF, Audrey S, Barker M, et al. Process evaluation of complex interventions: Medical Research Council guidance. *BMJ* 2015;350:h1258.

32 Campbell NC, Murray E, Darbyshire J, et al. Designing and evaluating complex interventions to improve health care. *BMJ* 2007;334(7591):455-9.

33 Yardley L, Morrison L, Bradbury K, et al. The person-based approach to intervention development: application to digital health-related behavior change interventions. *J Med Internet Res* 2015;17(1):e30.

34 Byrskog U, Essen B, Olsson P, et al. 'Moving on' Violence, wellbeing and questions about violence in antenatal care encounters. A qualitative study with Somali-born refugees in Sweden. *Midwifery* 2016;40:10-7.

35 Ahrne M, Schytt E, Andersson E, et al. Antenatal care for Somali-born women in Sweden: Perspectives from mothers, fathers and midwives. *Midwifery* 2019;74:107-115.

36 Lionis C, Papadakaki M, Saridaki A, et al. Engaging migrants and other stakeholders to improve communication in cross-cultural consultation in primary care: a theoretically informed participatory study. *BMJ open* 2016;6(7):e010822.

For peer review only - http://bmjopen.bmj.com/site/about/guidelines.xhtml

For peer review only

37 Johnson CE, Ali SA, Shipp MP. Building community-based participatory research partnerships with a Somali refugee community. *Am J Prev Med* 2009;37(6 Suppl 1):S230-6.

38 Attride-Stirling J. Thematic networks: an analytic tool for qualitative research. *Qualitative Research* 2001;1(3):385-405.

39 Ekman I, Swedberg K, Taft C, et al. Person-centered care - ready for prime time. *Eur J Cardiovasc Nurs* 2011;10(4):248-51.

40 Lundahl B, Moleni T, Burke BL, et al. Motivational interviewing in medical care settings: a systematic review and meta-analysis of randomized controlled trials. *Patient Educ Couns* 2013;93(2):157-68.

41 Wagner CC. Motivational Interviewing in Groups. New York: Guilford Publications; 2013.

42 Sharma J, O'Connor M, Rima Jolivet R. Group antenatal care models in low- and middle-income countries: a systematic evidence synthesis. *Reprod Health.* 2018;15(1):38.

43 Catling CJ, Medley N, Foureur M, Ryan C, Leap N, Teate A, Homer CS. Group versus conventional antenatal care for women. Cochrane Database Syst Rev. 2015;4;(2).

44 Gagnon AJ, DeBruyn R, Essen B, et al. Development of the Migrant Friendly Maternity Care Questionnaire (MFMCQ) for migrants to Western societies: an international Delphi consensus process. *BMC Pregnancy Childbirth* 2014;14:200.

45 Green JM, Kafetsios K, Statham HE, et al. Factor structure, validity and reliability of the Cambridge Worry Scale in a pregnant population. *J Health Psychol* 2003;8(6):753-64.

46 Cox J, Holden J, Henshaw C. Perinatal Mental Health. The Edinburgh Postnatal Depression Scale (EPDS) Manual. 2nd ed: The Royal College of Psychiatrists; 2012.

47 Sudman S, Bradburn NM, Schwarz N. Thinking about answers: the application of cognitive processes to survey methodology. San Francisco: Jossey-Bass Publishers; 1996.

48 Colon-Burgos JF, Colon-Jordan HM, Reyes-Ortiz VE, et al. Disparities and barriers encountered by immigrant Dominican mothers accessing prenatal care services in Puerto Rico. *J Immigr Minor Health* 2014;16(4):646-51.

49 Center for Disease Control and Prevention. Pregnancy Risk Assessment Monitoring System (PRAMS) [Internet]. [Accessed 2016 April 2]. Available from: http://www.cdc.gov/prams/.

50 Liamputtong P. Performing Qualtiative Cross-cultural Research. Cambridge: Cambridge University Press; 2010.

51 Hildingsson I, Rådestad I. Swedish women's satisfaction with medical and emotional aspects of antenatal care. *J Adv Nurs* 2005;52:239-249

For peer review only

52 Rubertsson C, Wickberg B, Gustavsson P, Radestad I. Depressive symptoms in early pregnancy, two months and one year postpartum-prevalence and psychosocial risk factors in a national Swedish sample. *Arch Women Ment Health* 2005;8(2):97-104.

53 Elo S, and Kyngas, H. The qualitative content analysis process. *J Adv Nurs* 2008 62(1):107-115.

For peer review only

For peer review only - http://bmjopen.bmj.com/site/about/guidelines.xhtml

For peer review only

For peer review only - http://bmjopen.bmj.com/site/about/guidelines.xhtml
